# Calcium orthophosphate coatings, films and layers

**DOI:** 10.1186/2194-0517-1-1

**Published:** 2012-09-26

**Authors:** Sergey V Dorozhkin

**Affiliations:** Kudrinskaja sq. 1-155, Moscow, 123242 Russia

**Keywords:** Calcium orthophosphates, Hydroxyapatite, Coatings, Layers, Films, Surface, Interface

## Abstract

In surgical disciplines, where bones have to be repaired, augmented or improved, bone substitutes are essential. Therefore, an interest has dramatically increased in application of synthetic bone grafts. As various interactions among cells, surrounding tissues and implanted biomaterials always occur at the interfaces, the surface properties of the implants are of the paramount importance in determining both the biological response to implants and the material response to the physiological conditions. Hence, a surface engineering is aimed to modify both the biomaterials, themselves, and biological responses through introducing desirable changes to the surface properties of the implants but still maintaining their bulk mechanical properties. To fulfill these requirements, a special class of artificial bone grafts has been introduced in 1976. It is composed of various mechanically stable (therefore, suitable for load bearing applications) biomaterials and/or bio-devices with calcium orthophosphate coatings, films and layers on their surfaces to both improve interactions with the surrounding tissues and provide an adequate bonding to bones. Many production techniques of calcium orthophosphate coatings, films and layers have been already invented and new promising techniques are continuously investigated. These specialized coatings, films and layers used to improve the surface properties of various types of artificial implants are the topic of this review.

**Electronic supplementary material:**

The online version of this article (doi:10.1186/2194-0517-1-1) contains supplementary material, which is available to authorized users.

## Review

### Introduction

All available materials have the specific characteristics of their own, namely, some of them are corrosive or biologically incompatible; some are sensitive to light or oxidation; some are hydrophilic or hydrophobic in nature, *etc.* Due to these reasons, various approaches have been already developed to modify the basic properties of diverse materials, and applying surface coatings, films or layers is a choice of option to solve some problems in a conventional form. For the particular case of artificial bone grafts, synthetic materials which are to be used in biological environments must display an adequacy of both their surface and bulk characteristics in order to fulfill the dual requirements of biocompatibility and suitable mechanical properties for the given application. Otherwise, due to a poor biocompatibility of improper compounds, fibrous tissues always encapsulate the implants made from such materials, which prolong the healing time. Considering that surface is always the first part of any insert that interacts with the host, various types of surface modifications have been developed to enhance biocompatibility and osteoconductivity of the implants (Ruckenstein and Gourisankar 1986).

On the other hand, it is well known that, due to the great chemical similarity to the inorganic part of bones and teeth of mammals, calcium orthophosphates (listed in Table 1) appear to be very friendly substances for the *in vivo* applications (Dorozhkin 2009, 2011; LeGeros 1991; Elliott 1994; Brown and Constantz 1994; Amjad 1997; Brès and Hardouin 1998; Chow and Eanes 2001; Hughes et al 2002; Dorozhkin 2012). However, since calcium orthophosphate bulk materials have a ceramic nature, they are mechanically weak (brittle); therefore, they cannot be subjected to the physiological loads as encountered in human skeletons, other than compressive ones. Therefore, for many years, the clinical applications of calcium orthophosphates alone have been largely limited to non-load bearing parts of the skeleton due to their inferior mechanical properties. Attempting to combine the advantages of various materials, which is one of the major innovations over the last approximately 40 years, researchers started to deposit biocompatible calcium orthophosphates onto the surface of mechanically strong but bio-inert or bio-tolerant materials (Ong and Chan 1999; de Groot et al. 1998; Campbell 2003; Kokubo 2008). For example, metallic implants are encountered in endoprosthesis (such as total hip joint replacements) and artificial teeth sockets because the requirements for a sufficient mechanical stability necessitate the use of a metallic body for such devices. As metals do not undergo bone bonding, *i.e.*, do not form mechanically stable links between the implant and bone tissues, they are coated by calcium orthophosphates exhibiting the bone-bonding ability between the metal and bone. After being implanted, calcium orthophosphate coatings, films and layers might be replaced by autologous bone because such coatings, films and layers participate in bone remodeling responses similar to natural bones (Ong and Chan 1999; Onoki and Hashida 2006; Kobayashi et al. 2007; Epinette and Geesink 1995; Willmann 1999; Schliephake et al. 2006; Kokubo et al. 2003; Habibovic et al. 2005; Hahn et al. 2009). Minimal requirements for HA coatings, films or layers (Table 2) have first been described in 1992 in the Food and Drug Administration (FDA) guidelines (Callahan et al. 1994), as well as a little bit later in the ISO standards (1996). Afterwards, the FDA guidelines were updated in 1997 (U.S. FDA 1995), while the ISO standards were updated in (ISO 2000) and (ISO 2008).

**Table 1:** Existing calcium orthophosphates and their major properties (Dorozhkin 2009, 2011)

Ca/P molar ratio	Compound	Formula	Solubility at 25°C -(-log(K_s_))	Solubility at 25°C (g/L)	pH stability range in aqueous solutions at 25°C
0.5	Monocalcium phosphate monohydrate (MCPM)	Ca(H_2_PO_4_)_2_· H_2_O	1.14	approximately 18	0.0 to 2.0
0.5	Monocalcium phosphate anhydrous (MCPA or MCP)	Ca(H_2_PO_4_)_2_	1.14	approximately 17	^a^
1.0	Dicalcium phosphate dihydrate (DCPD), mineral brushite	CaHPO_4_· 2H_2_O	6.59	approximately 0.088	2.0 to 6.0
1.0	Dicalcium phosphate anhydrous (DCPA or DCP), mineral monetite	CaHPO_4_	6.90	approximately 0.048	^a^
1.33	Octacalcium phosphate (OCP)	Ca_8_(HPO_4_)_2_(PO_4_)_4_· 5H_2_O	96.6	approximately 0.0081	5.5 to 7.0
1.5	α-Tricalcium phosphate (α-TCP)	α-Ca_3_(PO_4_)_2_	25.5	approximately 0.0025	^b^
1.5	β-Tricalcium phosphate (β-TCP)	β-Ca_3_(PO_4_)_2_	28.9	approximately 0.0005	^b^
1.2 to 2.2	Amorphous calcium phosphates (ACP)	Ca_*x*_H_*y*_(PO_4_)_*z*_· *n* H_2_O, *n* = 3 to 4.5%; 15 to 20% H_2_O	^c^	^b^	approximately 5 to 12 ^d^
1.5 to 1.67	Calcium-deficient hydroxyapatite (CDHA or Ca-def HA)^e^	Ca_10–*x*_(HPO_4_)_*x*_(PO_4_)_6–*x*_(OH)_2–*x*_ (0 < *x* < 1)	approximately 85	approximately 0.0094	6.5 to 9.5
1.67	Hydroxyapatite (HA, HAp or OHAp)	Ca_10_(PO_4_)_6_(OH)_2_	116.8	approximately 0.0003	9.5 to 12
1.67	Fluorapatite (FA or FAp)	Ca_10_(PO_4_)_6_ F_2_	120.0	approximately 0.0002	7 to 12
1.67	Oxyapatite (OA, OAp or OXA)^f^	Ca_10_(PO_4_)_6_O	approximately 69	approximately 0.087	^b^
2.0	Tetracalcium phosphate (TTCP or TetCP), mineral hilgenstockite	Ca_4_(PO_4_)_2_O	38 to 44	approximately 0.0007	^b^

^a^ Stable at temperatures above 100°C.

^b^ These compounds cannot be precipitated from aqueous solutions.

^c^ Cannot be measured precisely. However, the following values were found: 25.7 ± 0.1 (pH = 7.40), 29.9 ± 0.1 (pH = 6.00), 32.7 ± 0.1 (pH = 5.28). The comparative extent of dissolution in acidic buffer is ACP > > α-TCP > > β-TCP > CDHA > > HA > FA.

^d^ Always metastable.

^e^ Occasionally, it is called “precipitated HA”.

^f^ Existence of OA remains questionable.

**Table 2:** **FDA requirements for HA coatings (** Callahan et al. 1994**)**

Properties	Specification
Thickness	Not specific
Crystallinity	62% minimum
Phase purity	95% minimum
Ca/P atomic ratio	1.67 to 1.76
Density	2.98 g/cm^3^
Heavy metals	< 50 ppm
Tensile strength	> 50.8 MPa
Shear strength	> 22 MPa
Abrasion	Not specific

### General knowledge on coatings, films and layers

According to Wikipedia, the free encyclopedia, ‘Coating is a covering that is applied to the surface of an object, usually referred to as the substrate. In many cases, coatings are applied to improve surface properties of the substrate, such as appearance, adhesion, wettability, corrosion resistance, wear resistance and scratch resistance. In other cases, in particular, in printing processes and semiconductor device fabrication (where the substrate is a wafer), the coating forms an essential part of the finished product.’ (2012a). Obviously, all the aforementioned is also valid for films. A layer is another important definition. It is determined as a single thickness of some material covering a surface or forming an overlying part or segment.

Historically, involvement with coatings, films and layers dates to the metal ages of antiquity. Consider the ancient craft of gold beating and gilding, which has been practiced continuously for, at least, 4 millennia. The Egyptians appear to have been the earliest practitioners of this art. Many magnificent examples of statuary, royal crowns and coffin cases that have survived intact attest to the level of skills achieved. For example, leaf samples from Luxor dating to the Eighteenth Dynasty (1567 to 1320 BC) appear to be approximately 0.3-μm thick. Such leaves were carefully applied and bonded to smoothed wax or resin-coated wood surfaces in a mechanical (cold) gilding process to create the earliest coatings (Ohring 2002). Concerning the subject of this review, to the best of my findings, the first research paper on calcium orthophosphate coatings was published in 1976 (Sudo et al. 1976).

In spite of the fact that the technology of coatings, films and layers appears to be simultaneously one of the oldest arts and one of the newest sciences, the distinction among the coatings, films and layers is not well established yet; moreover, it may vary depending on the field of science and/or technology. For example, in food industry, the following statement has been published: ‘An edible coating (EC) is a thin layer of edible material formed as a coating on a food product, while an edible film (EF) is a preformed, thin layer, made of edible material, which once formed can be placed on or between food components (McHugh 2000). The main difference between these food systems is that the ECs are applied in liquid form on the food, usually by immersing the product in a solution-generating substance formed by the structural matrix (carbohydrate, protein, lipid or multi-component mixture), and EFs are first molded as solid sheets, which are then applied as a wrapping on the food product’. (Falguera et al. 2011). To clarify this topic further, an extensive search in the scientific databases (Scopus, ISI Web of Knowledge) has been performed, and a great number of fixed collocations have been revealed. For example, according to Scopus (as of May 2012), a combination of words ‘wear-protecting + coating’ in the publication titles is used more frequently if compared with that of ‘wear-protecting + film’ (75 and 11 publications, respectively). On the contrary, a combination of words ‘ferroelectric + film’ in the publication titles is used much more frequently if compared with that of ‘ferroelectric + coating’ (5,861 and 28 publications, respectively). Concerning the subject of current review, a combination of words ‘apatite + coating’ is found in the titles of 2,635 publications, while those of ‘apatite + film’ and ‘apatite + layer’ are found in the titles of 427 and 370 publications, respectively. A similar correlation is valid for the combinations of words ‘calcium + phosphate + coating’, ‘calcium + phosphate + film’ and ‘calcium + phosphate + layer’ (they are found in the titles of 737, 138 and 149 publications, respectively). Therefore, both HA and all other calcium orthophosphates are most commonly associated with coatings. Perhaps, the aforementioned facts might be just a matter of terminology or even a habit for each particular sub-direction of science and technology.

Now it is necessary to classify various types of coatings, films and layers. In general, many possibilities are available. For example, they might be classified according to their structural material, such as metallic, polymeric, ceramic or composite coatings, films and layers. Furthermore, they might be classified according to their properties, such as biodegradability, edibility, transparency, reflectivity, conductivity, hardness, porosity, solubility, permeability, etc*.*, as well as by the adhesion strength to various substrates. Besides, using a formation approach, all coatings, films and layers can be divided into two big categories: i) conversion ones, which are formed by reaction products of the base material (for example, formation of an oxide layer by surface oxidation) and ii) deposited ones. In turn, the deposited coatings, films and layers might be further classified according to the deposition techniques (Table 3). More to the point, since coatings and films may consist of either one or many individually deposited layers, all of them might be divided into monolayer coatings and films, and multilayer ones. While the former ones are produced by a single stage, the latter ones are produced by layer-by-layer deposition techniques. Furthermore, the individual layers of the multilayer coatings and films might be both indistinguishable from each other (in this case, the multilayer coatings and films behave as a thick monolayer) and distinguishable from each other. In the latter case, there might be an opportunity (sometimes, only hypothetical) to remove one or several individual layers from the surface, making coatings and films thinner. Finally yet importantly, all types of layers, coatings and films might be thin or thick. These terms appear to be relative, and the distinction between them is not well determined either; furthermore, it depends on the specific application. Nevertheless, in general, researchers consider a thin layer, film or coating as one ranging from fractions of a nanometer to several micrometers in thickness. Therefore, a thick layer, film or coating has thickness exceeding several micrometers. Interestingly that, according to the aforementioned scientific databases, all types of coatings, films and layers are much more often ‘thin’ than ‘thick’, namely, according to Scopus (as of May 2012), a combination of words ‘thin + coating’ in publication titles is used more frequently if compared with that of ‘thick + coating’ (2,608 and 468 publications, respectively). Similarly, a combination of words ‘thin + film’ in publication titles is used much more frequently if compared with that of ‘thick + film’ (144,106 and 7,077 publications, respectively), and a combination of words ‘thin + layer’ in publication titles is used much more frequently if compared with that of ‘thick + layer’ (26,603 and 1,443 publications, respectively). Concerning the physical state of the precursor materials, layers, coatings and films may be applied as liquids, gasses or solids, which might be used as still another classification type. The quality of coatings, films and layers is usually assessed by measuring their porosity, chemical composition, homogeneity, macro- and micro-hardness, bond strength and surface roughness.

**Table 3:** **Various techniques to deposit bio-resorbable coatings, films and layers of calcium orthophosphates on metal implants (** Sun et al.^2001^; Yang et al. 2005; Narayanan et al. 2010**)**

Technique	Thickness	Advantages	Disadvantages
Thermal spraying	30 to 200 μm	High deposition rates; low cost	Line of sight technique; high temperatures induce decomposition; rapid cooling produces amorphous coatings; high temperatures prevent from simultaneous incorporation of biological agents
Plasma spraying	30 to 200 μm	High deposition rates; improved wear and corrosion resistance and biocompatibility	Line of sight technique; high temperatures induce decomposition; rapid cooling produces amorphous coatings; high temperatures prevent from simultaneous incorporation of biological agents
Magnetron sputtering	0.5 to 3 μm	Uniform coating thickness on flat substrates; high purity and high adhesion; dense pore-free coatings; excellent coverage of steps and small features; ability to coat heat-sensitive substrates	Line of sight technique; expensive; low deposition rates; produces amorphous coatings; high temperatures prevent from simultaneous incorporation of biological agents
Pulsed laser deposition (laser ablation)	0.05 to 5 μm	Coatings with crystalline and amorphous phases; dense and porous coatings; high adhesive strength	Line of sight technique; expensive; high temperatures prevent from simultaneous incorporation of biological agents
Ion beam deposition	0.05 to 1 μm	Uniform coating thickness; high adhesive strength	Line of sight technique; expensive; produces amorphous coatings
Dynamic mixing method	0.05 to 1.3 μm	High adhesive strength	Line of sight technique; expensive; produces amorphous coatings
Dip and spin coating	2 μm to 0.5 mm	Inexpensive; coatings applied quickly; can coat complex substrates	Requires high sintering temperatures; thermal expansion mismatch
Sol–gel technique	< 1μm	Can coat complex shapes; low processing temperatures; thin coatings; inexpensive process; can incorporate biological molecules	Some processes require controlled atmosphere processing; expensive raw materials
Electrophoretic deposition	0.1 to 2.0 mm	Uniform coating thickness; rapid deposition rates; can coat complex substrates; can incorporate biological molecules	Difficult to produce crack-free coatings; requires high sintering temperatures
Electrochemical (cathodic) deposition	0.05 to 0.5 mm	Good shape conformity; room temperature process; uniform coating thickness; short processing times; can incorporate biological molecules	Sometimes stressed coatings are produced, leading to their poor adhesion with substrate; requires good control of electrolyte parameters
Biomimetic process	< 30 μm	Low processing temperatures; can form bonelike apatite; can coat complex shapes; can incorporate biological molecules	Time consuming; requires replenishment and a pH constancy of the simulating solutions (HBSS, SBF, etc*.*)
Hot isostatic pressing	0.2 to 2.0 μm	Produces dense coatings	Cannot coat complex substrates; high temperature required; thermal expansion mismatch; elastic property differences; expensive; removal/interaction of encapsulation material; high temperatures prevent from simultaneous incorporation of biological agents
Micro-arc oxidation	3 to 20 μm	Simple, economical and environmentally friendly coating technique, suitable for coating of complex geometries	Except of calcium orthophosphates, coatings always contain admixture phases

HBSS, Hank's balanced salt solution; SBF, simulated body fluid.

Further, one should mention on the reasons why people apply layers, coatings and/or films to the surface of various materials. They are various, for example:

1. The core contains a material, which is toxic, provokes adverse responses, allergic reactions, etc., or has a bitter taste, an unpleasant odor, etc.;
2. Layers, coatings and/or films protect the core material from the surroundings to improve its stability and shelf life;
3. Layers, coatings and/or films develop the mechanical integrity, which means that coated products are more resistant to mishandling (abrasion, attrition, etc.);
4. To modify surface properties of the core, such as biocompatibility, light reflection, electrical conductivity, color, etc.;
5. Decoration (in the cases, when the core alone is inelegant);
6. The core contains a material, which migrates easily to stain hands, clothes and other objects;
7. To modify the release profile of active components, e.g*.*, drugs, from the core.

Reason numbers 1, 2, 3, 4 and 7 appear to be applicable to the biomedical field in general, while reason numbers. 1, 2 and 4 are relevant to the subject of this review.

To conclude this section, one should note that, in a certain sense, all types of coated materials resemble the functionally graded ones but with an extremely high gradient in both the composition and properties at the core/coating interface.

### Preparation

#### Brief knowledge on the important pre- and post-deposition procedures

**Figure 1:**
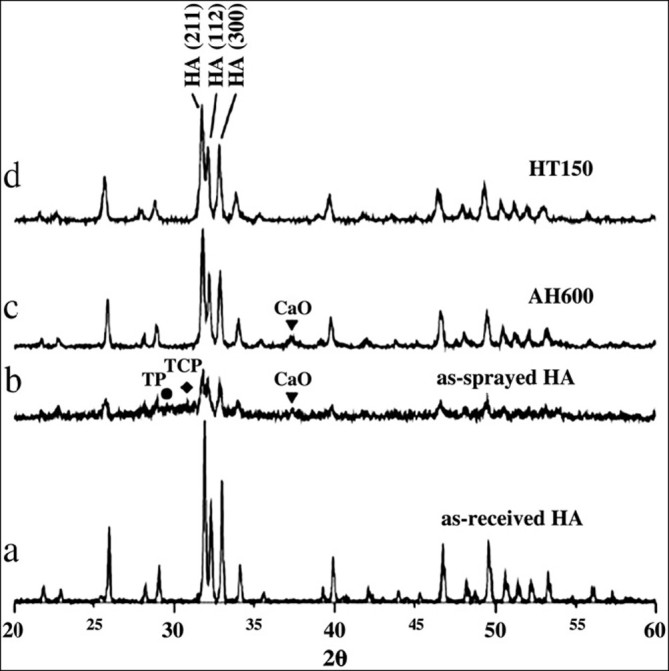
**The XRD patterns.** **(a)** an initial HA powder, **(b)** as sprayed HA coatings, **(c)** air heat-treated at 600°C HA coatings (AH600) and **(d)** hydrothermally treated at 150°C HA coatings (HT150). It is easily seen that both the crystallinity and phase purity of poorly crystalline HA coatings increased after both heat and hydrothermal treatments. Reprinted from Yang et al. (2009a) with permission.

Due to the unfavorable mechanical properties of bulk calcium orthophosphate bioceramics, an extensive research has been focused on the development of calcium orthophosphate coatings, films and layers on the surfaces of various materials. Various deposition techniques have been already proposed, which are discussed below. The major advantages and disadvantages of the available deposition techniques have been summarized in Table 3 (Sun et al. 2001; Yang et al. 2005; Narayanan et al. 2010). However, in the vast majority of the cases, prior to be coated, an object (a substrate) needs to be prepared for coating. This normally consists of some kind of cleaning (e.g., ultrasonically in an acetone or ethanol bath to remove dirt, oil and other contaminants adhering to the surface) and may include etching, tarnishing, grounding and/or application of a conversion coating (Chou and Chang 2001). Besides, various types of physical modifications of the surface, such as sand- (Cao et al. 2010a) or grit-blasting and polishing, as well as wetting or drying procedures might be applied as well. Furthermore, after calcium orthophosphate coatings, films and/or layers have been formed, various types of post-deposition treatments might be also necessary to improve their properties. For example, post-deposition heat treatment (annealing) of calcium orthophosphates leads to conversion of the deposited amorphous and non-apatite phases into HA with simultaneous increasing of coating crystallinity, enhancing corrosion resistance, as well as reducing the residual stress (Ji and Marquis 1993; Ong and Lucas 1994; Yoshinari et al. 1997; Erkmen 1999; Burgess et al. 1999; Sridhar et al. 2003; Yang et al. 2003a; Lee et al. 2005a; Johnson et al. 2006; Yang et al. 2009a). The annealing can be done by various ways, including laser treatment (Cannillo et al. 2009) or electric polarization in alkaline solutions (Huang et al. 2009). Furthermore, the presence of water during the post-deposition heat treatment also plays an important role in this conversion (Yang et al. 2009a; Cao et al. 1996; Yang et al. 2003b), namely, in comparison to heat treatments at 450°C in dry conditions, the presence of water vapor resulted in a significant increase in coating crystallinity (Yang et al. 2003b). Similar positive effect of hot water was obtained in other studies (Yang et al. 2009a; Saju et al. 2009; Ozeki et al. 2010) in which post-deposition hydrothermal treatment at 100°C to 170°C was used (Figure 1).

#### Thermal spraying techniques

Thermal spraying is the process in which melted, softened or heated materials are sprayed onto a surface to be deposited on it. A feedstock with a coating material or a coating precursor might be heated by various ways, such as a high temperature flame or a plasma jet, by means of which thermal spraying is classified into flame spraying and plasma spraying. The principal difference between these two techniques is the maximum temperature achievable. In general, thermal spraying provides thick (from approximately 20 μm to several millimeters, depending on the process and feedstock) coatings, films and layers over a large area at high deposition rate, as compared to other coating processes such as electroplating, physical and chemical vapor deposition (Table 3). Coating materials are fed in powder or wire form, heated to a molten or semi-molten state and accelerated towards substrates in the form of micrometer-size particles. Resulting coatings or films are formed by a continuous buildup of successive layers of liquid droplets, softened material domains and hard particles. For all types of thermal spraying techniques, the quality of coatings, layers and films is generally increased with increasing particle velocities (Fauchais et al. 2001).

Since thermal spraying occurs at very high temperatures, the substrates are heated up as well. In some cases, this might result in phase transformation and recrystallization of the near surface zones. For example, a martensitic transformation and recrystallization was found to occur in near surface of a low-modulus Ti-24Nb-4Zr-7.9Sn alloy substrate after application of a plasma-sprayed HA coating. Both phenomena were attributed to the combination of temperature with cooling process (Zhao et al. 2011). Certainly, such phenomena introduce additional ambiguities to the mechanical and adhesive properties of the deposited coatings, films and layers.

##### Plasma spraying

Plasma is often referred to as the fourth state of matter, as it differs from solid, liquid and gaseous states, and does not obey the classical physical and thermodynamic laws. Plasmas are used in many different processing techniques, for example, for modification and activation of various surfaces. Much research is currently being done to understand and control them (Freidberg 2007).

**Figure 2:**
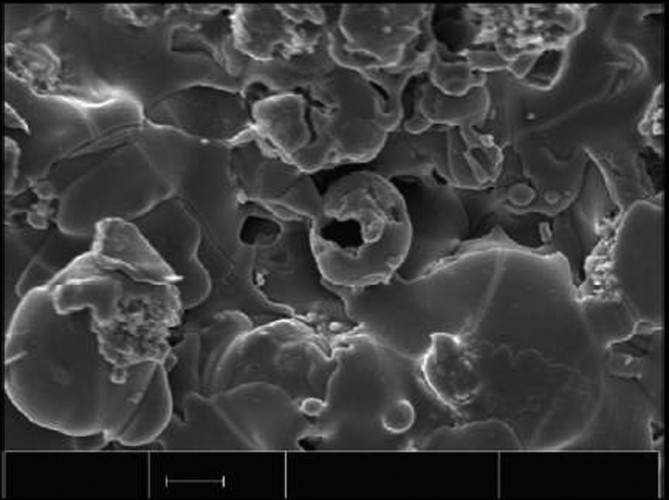
**A scanning electron microscopy of a typical plasma sprayed HA coating on titanium implants.** Bar is 10 μm. Reprinted from Layrolle (2011) with permission.

According to de Groot et al. (1998), a plasma spraying technique was discovered accidentally in 1970 by a student, who used the equipment to study melted and rapidly solidified aluminum oxide coatings on a metal substrate (Herman 1988). In plasma spraying, a material to be deposited (feedstock) - typically as a powder, sometimes as a liquid, suspension or wire - is introduced into a plasma jet, emanating from a plasma torch (other names: plasma arc or plasma gun) because a stream of gasses (usually, argon; however, helium, hydrogen or nitrogen might be used as well) passes through this torch. The torch turns these gasses into ionized plasma of a very high temperature (up to approximately 20,000 K) and with a high speed of up to 400 m/s. In the jet, the material is either melted or heat softened, and these molten or softened droplets flatten and propel towards a substrate to be deposited on it. Appropriate cooling techniques keep the temperature of the substrate below 100°C to 150°C. Since the temperature of the plasma rapidly decreases as a function of distance, the droplets rapidly solidify and form deposits. Commonly, the deposits consist of a multitude of pancake-like lamellae called ‘splats’, formed by flattening of the liquid or softened droplets. They remain adherent to the substrate as coatings, films and layers. As the feedstock typically consists of powders with sizes from several micrometers to approximately 1 mm, the lamellae have thickness in the micrometer range and lateral dimension from several to hundreds of micrometers. That is why thick coatings, films or layers might be produced only. One pass of the plasma gun can produce a layer of about 5 to 15-lamellae thick. Once a layer has been applied to the whole substrate, the gun returns to the initial position and another layer is applied (Fauchais 2004). Typical current values that are used for spraying HA coatings range from 350 A (Cao et al. 1996) to 1,000 A (Quek et al. 1999). Good schematic setups of the plasma spraying process are available in literature (Narayanan et al. 2010; Fauchais 2004; Paital and Dahotre 2009a; Layrolle 2011; Surmenev 2012). A typical image of a plasma-sprayed HA coating is shown in Figure 2 (Layrolle 2011).

Depending on the experimental conditions, various sub-modifications of plasma spraying technique have been outlined, namely atmospheric plasma spraying (Heimann 2006), vacuum (or low-pressure) plasma spraying (Gledhill et al. 1999, 2001), powder plasma spraying, suspension plasma spraying (Jaworski et al. 2009; Gross and Saber-Samandari 2009; Podlesak et al. 2010), liquid (or solution) plasma spraying (Huang et al. 2010) and, gas plasma spraying (Morks and Kobayashi 2007; Wu et al. 2009) techniques, and all of them are used to fabricate bioactive calcium orthophosphate-based coatings, films and layers. Such modifications have some specific advantages, *e.g.*, they allow obtaining thinner coatings of 5 to 50 μm, which are a few times thinner than those obtained by dry powder processing (Table 3) (Surmenev 2012). In addition, there is a microplasma spraying technique (Dey and Mukhopadhyay 2010; Dey et al. 2011; Dey and Mukhopadhyay 2011), which is characterized by small dimensions, a low level (25 to 50 dB) of noise and hardly any dust, as well as a low power consumption. All of these make it possible to operate under normal workroom conditions. The process provides deposition of coatings, films and layers on small-sized parts and components, including those with fine sections, this being unachievable with any other methods. Due to a low heat input of the microplasma jet, overheating of the powder particles as well as excessive local overheating of the substrate is reduced. The mechanical properties of the coatings, films and layers are generally good (Dey and Mukhopadhyay 2010; Dey et al. 2011; Dey and Mukhopadhyay 2011). This makes it possible to widen the application scales of plasma spraying and produce different functional coatings.

It is important to stress that, among the deposited lamellae, there are small voids, such as pores, cracks and regions of incomplete bonding. Due to such inhomogeneous structure, the deposits can have properties significantly different from the initial bulk materials (Fauchais 2004). In addition, due to both very high processing temperatures (leading to dehydroxylation of HA and partial decomposition of any other material) followed by rapid solidification, various admixtures and metastable phases are usually present in the deposits. For example, in the case of plasma spraying of calcium orthophosphates, complicated mixtures of various phases (high temperature ACP, α-TCP, β-TCP, HA, OA, TTCP) with other compounds, such as calcium pyrophosphates, calcium metaphosphates, CaO, etc. are obtained (Cao et al. 2010a; Zyman et al. 1993; Weng et al. 1993; Zyman et al. 1994; Tong et al. 1995; McPherson et al. 1995; Yang et al. 1995; Tong et al. 1998; Park et al. 1998; Gross et al. 1998a; Gross et al. 1998b; Heimann and Wirth 2006; Roy et al. 2011). Thus, the chemical and phase compositions of the final coatings, films and layers are dependent on the thermal history of the powder particles. This leads to variable solubility of the deposited coatings, films and layers, dictated by the amounts of more soluble phases, such as ACP. Furthermore, the distribution of by-product and metastable phases in the coatings, films and layers appears to be inhomogeneous. For example, the coating crystallinity was reported to be lower at the interface with the Ti substrate than at the surface of the coating. This happened because metals had a higher rate of thermal diffusivity than calcium orthophosphates and, thus, the cooling rate of the first layer was faster (Gross et al. 1998b). Besides, residual stresses in the plasma sprayed coatings, films and layers were found and measured (Valter et al. 1997; Tsui et al. 1998; Yang et al. 2000; 2003c; Yang and Chang 2005; Carradó 2010; Yang 2011).

**Figure 3:**
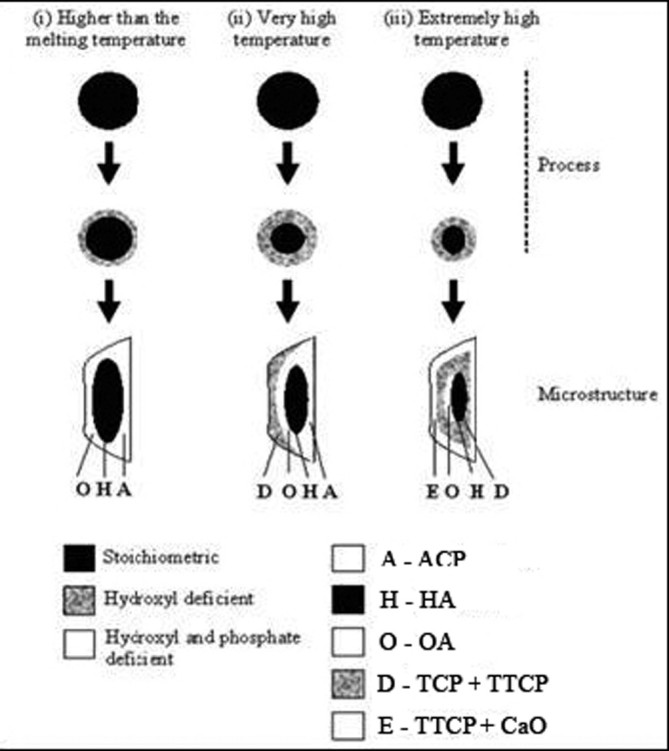
**A proposed model for phase formation in the plasma sprayed HA coatings.** The process stage depicts the various melt chemistries as a function of particle temperature. The microstructure depicts the different phases that can be formed in a lamella. Reprinted from Khor and Cheang (1993) with permission.

**Figure 4:**
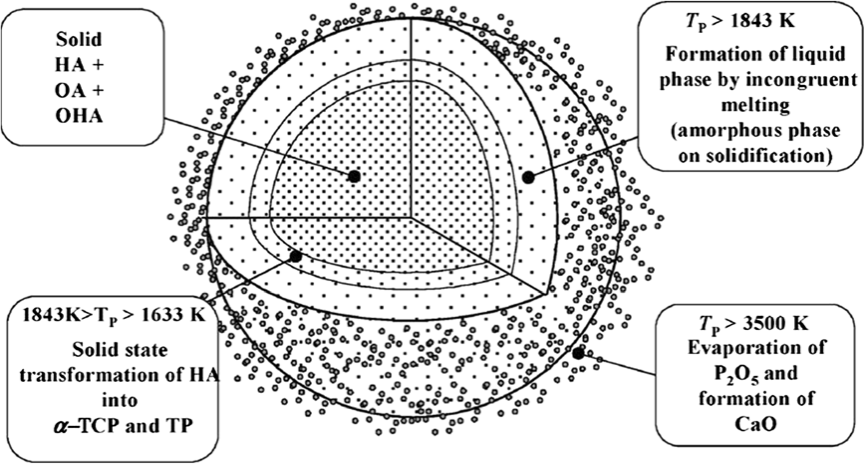
**Temperature fields of HA powder particle at impact and assumed phase transformations.** Reprinted from Dyshlovenko et al. (2004) with permission.

A diagram for the formation of various phases during plasma spraying of HA coatings is presented in Figure 3 (Khor and Cheang 1993). According to the authors, if the outside skin of an HA particle is molten and the core remains un-molten, insufficient heat is transferred to melt the particle completely. This model is modified to a totally molten hydroxyl-rich core (with the stoichiometry of HA) with further changes depending on the heat transfer to the particle. The first condition depicts a molten droplet with a hydroxyl-depleted skin. The center containing the hydroxyl-rich molten material will crystallize upon deposition to form HA. The dehydroxylated region, which is exposed to the substrate upon droplet spreading, will form an ACP phase, but the area distant from the substrate will crystallize to form OA. OA requires smaller atomic rearrangements to occur for crystallization from a viscous melt, and, therefore, crystallizes in preference to a mixture of TCP and TTCP. Growth of HA will begin in the hydroxyl-rich core and will finally change to OA in response to the depleted hydroxyl concentration at the top of the lamellae (Figure 3, case (i)). If the molten particle flattens to an extent where the cooling rate is increased, then the entire particle becomes amorphous. Both TCP and TTCP are observed in greater quantities when a higher heat transfer to the particle prevails. If the heat dissipation is slow through the already-solidified amorphous and crystalline layers of the coating, TCP and TTCP can be nucleated at the top surface of the lamellae (Figure 3, case (ii)). The growth of TCP and TTCP may delay the growth of OA with the latent heat of fusion. With a high level of dehydroxylation in the molten particle, lesser amounts of HA or OA will form, and so the large volume of dehydroxylated material will then mostly contain TCP and TTCP. The growth mechanism may begin within the droplet, since a more fluid droplet facilitates faster diffusion. Calcium oxide is observed when even higher heating conditions are employed. In addition to being hydroxyl deficient, the outer shell of the molten particle also becomes phosphate deficient (Figure 3, case (iii)) (Narayanan et al. 2010). In addition, a numerical simulation model of HA powder behavior in plasma jet was suggested (Dyshlovenko et al. 2004). Within this model, the authors created temperature fields inside an HA particle before impact and their transformation into crystal phases after rapid solidification and cooling (Figure 4).

There are a large number of technological parameters that influence the interaction of the particles with the plasma jet and the substrate and, therefore, the properties of final coatings, films and layers. These parameters include feedstock type, plasma gas composition, flow rate, energy input, torch offset distance, substrate cooling, etc. (Cizek et al. 2007). Furthermore, due to the very high temperatures of plasmas, the aforementioned thermodynamic instability of calcium orthophosphates at such temperatures plays an important role in the final properties of the deposited coatings, films and layers. Ideally, only a thin outer layer of each powder particle should be heated to the molten plastic state, which unavoidably undergoes both chemical transformations and phase transitions. This plastic state is necessary to ensure dense and adhesive coatings but it should comprise just a negligible volume fraction of the particles. By choosing optimum relations among particle size, type of gas, speed of the plasma and cooling process of the coated surface, one obtains calcium orthophosphate coatings films and layers with the desired thickness and crystallinity (de Groot et al. 1987; Cook et al. 1988; Stevenson et al. 1989; Wolke et al. 1992; Sun et al. 2003; Prymak et al. 2004).

The dimensions of calcium orthophosphate particles were found to affect their melting characteristics within the plasma flame, namely, large particles undergo a lesser degree of melting in the plasma flame than small particles (Cheang and Khor 1995; Kweh et al. 2000). For example, during spraying of HA particles with dimensions exceeding approximately 55 μm they were found to remain crystalline and showed little or no melting during plasma spraying. HA particles with dimensions within 30 to 55 μm were partially melted and consisted of mixtures of crystalline and amorphous phases, while HA particles less than approximately 30 μm were fully melted and contained large amounts of ACP and traces of CaO (Cheang and Khor 1995). In another study, plasma sprayed HA particles of 20 to 45 μm in size were found to produce denser lamellar coatings than the coatings obtained by plasma spraying of 45 to 75 and 75 to 125-μm HA particles. Coatings formed from 20 to 45-μm sized HA particles did not show the presence of cavities but contained a flatter smoother surface profile as a result of neatly stacked disk-like splats, while coatings formed from 45 to 75 and 75 to 125-μm sized HA particles contained numerous un melted particles, cavities and macropores (Kweh et al. 2000). Interestingly, the coating roughness might be used as a measure of the melting degree of particles within the plasma flame, namely, when the particles reach a more fluid state within the plasma flame, they become less viscous and can be spread out to a greater degree on impact with the substrate. A smoother coating will result in this case. Partially melted particles will not be able to flatten on the coating surface. This situation will lead to large undulations and a rough coating (Gross and Babovic 2002).

Further details on the plasma spraying technique are available in excellent reviews (Surmenev 2012).

##### High velocity oxy-fuel spraying

In 1990s, a new class of thermal spray processes called high velocity oxy-fuel (HVOF) spraying was developed (Oguchi et al. 1992; Sobolev and Guilemany 1996). A mixture of gaseous (hydrogen, methane, propane, propylene, acetylene, natural gas, etc.) or liquid (kerosene, etc.) fuel and oxygen is fed into a combustion chamber, where they are ignited and combusted continuously. The resultant hot gas at a pressure approximately 1 MPa emanates through a converging-diverging nozzle and travels through a straight section. The jet velocity (> 1,000 m/s) at the exit of the barrel exceeds the speed of sound. A powder feed stock is injected into the gas stream, which accelerates the powder up to 800 m/s. The stream of hot gas and powder is directed towards the surface to be coated. The powder partially melts in the stream and deposits upon the substrate. The resulting calcium orthophosphate coatings, layers and films have a low porosity and a high adhesion strength (Oguchi et al. 1992; Sobolev and Guilemany 1996; Haman et al. 1999; Li et al. 2000, 2002a; Khor et al. 2003a, 2003b, 2004; Hasan and Stokes 2011).

**Figure 5:**
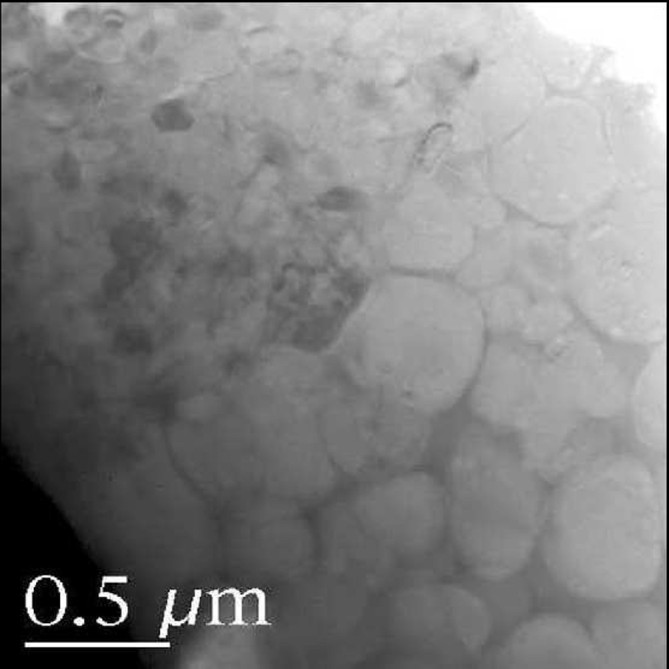
**TEM image of as-sprayed HA coating.** This show the interface between unmelted and un-melted parts within a HA splat and different grain size. Reprinted from Khor et al. (2003b) with permission.

Similar to the aforementioned results on plasma spraying, in the case of HVOF spraying, larger particles of calcium orthophosphates were also found to undergo a lesser degree of melting than smaller particles (Khor et al. 2003b), namely, cross-sectional SEM investigations of the sprayed HA particles of 50 ± 10 μm in sizes revealed that they were melt only partially from the surface, while those for HA particles of 30 ± 10 μm in sizes revealed that they were melt almost completely. The coating morphology shown in Figure 5 further reveals the influence of the melt state on grain size of the coatings. It clearly demonstrates the interface zone between the melted and un melted parts within a HA splat. It is noted that the HA grains located in un melted part are of far larger size than those in melted part, which states the influence of rapid cooling on grain growth during coating formation (Khor et al. 2003b). Furthermore, Raman spectroscopy qualitative inspection on the sprayed HA particles (partially melted) revealed that a thermal decomposition of HA occurred within the melted part rather than the unmelted zone (Khor et al. 2004). Therefore, to both achieve high crystallinity of the coatings and reduce the amount of admixture phases, the appropriate powder size together with the apt HVOF spray parameters must be carefully selected.

#### Wet techniques

As follows from the definition, all types of wet deposition techniques occur from either solutions or suspensions both aqueous and non-aqueous. Furthermore, all of them occur at moderate temperatures (Nijhuis et al. 2010). Depending on the solution pH, various calcium orthophosphates might be precipitated (Table 1) and, therefore, be deposited as coatings, films and layers. In general, the deposition kinetics depends on the solution supersaturation, concentration of the reagents, temperature, presence or absence of admixtures, nucleators, inhibitors, etc. As to the precipitation mechanism of calcium orthophosphates from aqueous solutions, this process appears to be rather complicated; for the biologically relevant calcium orthophosphates (OCP, CDHA and HA), the crystallization process occurs via formation of one or several intermediate and/or precursor phases, such as ACP, DCPD and/or OCP. The detailed description of the precipitation mechanisms of various calcium orthophosphates is beyond the scope of current review; the interested readers are referred to the special literature on the topic (Wang and Nancollas 2008; Wang et al. 2011).

For some types of the wet techniques, specific surface preparation techniques appear to be necessary. For example, if calcium orthophosphates need to be deposited on titanium or its alloys, a surface layer of hydrated titanium hydroxides should be created prior the deposition (Wang et al. 2008a). This can be done by various oxidation techniques, such as alkali treatment (de Andrade et al. 2002; Liang et al. 2003; Wang et al. 2000), oxidation in H_2_O_2_ (Wang et al. 2000), micro-arc oxidation (Song et al. 2004), pre-calcification in boiling Ca(OH)_2_ solution (Wen et al. 1997; Chen et al. 2009a) or using water vapor treatment (Feng et al. 2002). Positive effects of the presence of hydrated silica (Li et al. 1994) and sodium (Pham et al. 2002) on the surface are known as well. Since the detailed description of the surface preparation of metals is beyond the scope of this review, the interested readers are referred to the special literature on the subject (Narayanan et al. 2010; Nanci et al. 1998; Liu et al. 2004; Rautray et al. 2010; Variola et al. 2011).

##### Electrophoretic deposition

According to Wikipedia, the free encyclopedia. ‘Electrophoretic deposition is a term for a broad range of industrial processes, which includes electrocoating, e-coating, cathodic electrodeposition and electrophoretic coating or electrophoretic painting.’ (2012b). A characteristic feature of this process is that charged colloidal particles suspended in a liquid medium migrate under the influence of a direct current electric field (electrophoresis) and are deposited onto a conductive substrate of the opposite charge (Besra and Liu 2007).

Since electrophoretic deposition is designed to apply materials to any electrically conductive surface, it is used to achieve calcium orthophosphate coatings, layers or films on various metallic substrates only (Ducheyne et al. 1986, 1990; Zhitomirsky and Gal-Or 1997; Han et al. 1999a; Zhitomirsky 2000; Wei et al. 2001; Stoch et al. 2001; Wang et al. 2002; de Sena et al. 2002; Ma et al. 2003a; Mondragón-Cortez and Vargas-Gutiérrez 2004; Meng et al. 2006, 2008). This approach is especially useful for porous metallic structures. To create coatings, layers or films, calcium orthophosphate powders are suspended in water or other suitable liquids to produce a coating bath, followed by deposition onto a metallic surface. The proper dimensions of the particles to be deposited are very important because the particles must be fine enough to remain in suspension during the coating process. Electrophoretic deposition normally involves submerging a metallic substrate into a container or vessel, which holds the coating bath, and applying direct current electricity using electrodes, where the substrate is one of the electrodes (anode or cathode). An applied electric field is the driving force of the deposition (Besra and Liu 2007). Depending on the mode and sequence of voltage applied, electrophoretic deposition of calcium orthophosphates can be carried out at either constant (Meng et al. 2006) or dynamic (Meng et al. 2008) voltage.

After deposition, an object is normally rinsed off to remove the undeposited bath, followed by sintering in a high vacuum (10^–6^ to 10^–7^ Torr) at 850°C to 950°C (Besra and Liu 2007). The resulting coatings, layers or films consist of a number of calcium orthophosphate phases plus various random admixtures. For example, in the case of electrophoretically deposited CDHA coatings, the sintering results in their transformation to biphasic (HA + β-TCP) coatings (Han et al. 1999a). Their thicknesses can be varied by changing the electrical field strength and the deposition time. Further, at the coating/substrate interface various metal-phosphorus compounds might be formed due to mutual inter-diffusion of calcium orthophosphates and atoms of the metallic substrate. Unfortunately, due to densification during sintering, shrinkage and cracking of the coatings, layers or films can occur. In addition, thermal stresses induced by the differences in thermal expansion coefficients between the core and the coating during sintering and cooling can lead to cracking (de Groot et al. 1998).

The surface morphology of the electrophoretically deposited calcium orthophosphate coatings was found to depend on applied voltage (Mondragón-Cortez and Vargas-Gutiérrez 2004), deposition time (Mondragón-Cortez and Vargas-Gutiérrez 2004) and powder concentration (Meng et al. 2006), namely, at 200 V, the deposited particles had dimensions within 0.20 to 0.35 μm; at 400 V, the particle size range increased up to 0.35 to 0.80 μm and at 800 V, the particle size range increased up to 0.80 to 1.20 μm. Furthermore, increasing voltages resulted in increasing of the amount of deposited calcium orthophosphates. Besides, porous and roughened coatings were obtained at a higher electric field, while dense coatings of finer particle size were obtained at a lower electric field (Mondragón-Cortez and Vargas-Gutiérrez 2004). Similar effect was noticed for the deposition time: the shorter the time, the smaller particles were deposited (Mondragón-Cortez and Vargas-Gutiérrez 2004). Concerning the powder concentration in suspensions, for a low HA concentration, the coatings were very rough, and a great level of agglomeration was noticed. At higher HA concentrations, the coatings became uniform and crack free, and there was less agglomeration. At very high concentrations of HA, many cracks were found (Meng et al. 2006). These results indicate that powder concentration, deposition time and applied potential have a significant effect on the coating morphology.

Interestingly, some specific types of calcium orthophosphate bioceramics might be prepared by electrophoretic deposition (Zhitomirsky 2000; Wang et al. 2002; Ma et al. 2003b). For example, hollow HA fibers of various diameters were fabricated (Zhitomirsky 2000). In the first step, submicron HA powders were electrophoretically deposited on individual carbon fibers, carbon fibers bundles and felts. Then, they were burned out and sintered to remove the carbon substrate and leave behind the corresponding ceramic replicas (Zhitomirsky 2000). Similarly, uniform HA tubes were prepared by electrophoretic deposition of HA powders on carbon rods by repeated depositions at room temperature (Wang et al. 2002). The repeated deposition process was necessary to produce thicker multilayered coatings with no surface cracks. The green bodies were then sintered under a range of temperatures varying from 1,150°C to 1,300°C to burn out carbon and obtain HA tubes (Wang et al. 2002). Furthermore, porous calcium orthophosphate scaffolds were fabricated by electrophoretic deposition (Ma et al. 2003b).

To conclude, electrophoretically deposited calcium orthophosphate coatings on implants are commercially available. The examples include BIONIT® (DOT GmbH, Rostock, Germany) and BoneMaster® (BIOMET Corp., Warsaw, IN, USA) (Layrolle 2011). In addition, various modifications and hybrid technologies, such as plasma-assisted electrophoretic deposition (Nie et al. 2001) and a combination of micro-arc oxidation with electrophoresis (Nie et al. 2000) have been developed as well.

##### Electrochemical (cathodic) deposition

In electrochemical deposition of calcium orthophosphates, a supersaturated or a metastable aqueous electrolyte containing calcium and orthophosphate ions is used. Various electrochemical reactions occurring in the electrolyte near electrodes induce local pH increase, and thus, calcium orthophosphate crystals are nucleated and grow on the electrodes (Manso et al. 2000; Duan et al. 2003; Lu et al. 2005). Obviously, only conductive materials might be coated by this technique. A typical setup includes a platinum electrode (anode) and a metallic implant (cathode) connected to a current generator. Since electrochemical deposition usually occurs on the negatively charged electrodes, in literature it is sometimes referred to as cathodic deposition (Zhao et al. 2003; Blackwood and Seah 2009; Roguska et al. 2011). The electrochemical reactions occurring with the ions during the deposition of calcium orthophosphates might be found in literature (Kuo and Yen 2002; Yen and Lin 2002).

**Figure 6:**
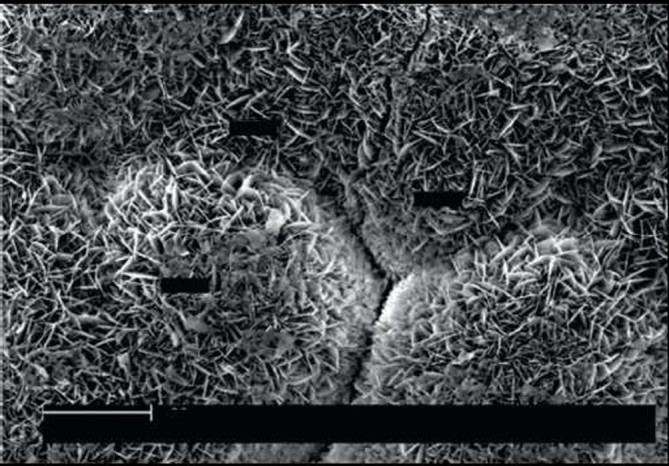
**A scanning electron microscopy of a typical electrochemically deposited coating on titanium.** Bar is 20 μm. Reprinted from Layrolle (2011) with permission.

Since electrochemical deposition of calcium orthophosphate coatings, films and layers occurs from aqueous solutions, it is commonly performed at ambient conditions (Rossler et al. 2003; Lin et al. 2003). However, electrochemical deposition performed in an autoclave at 80°C to 200°C is also known (Ban and Maruno 1998). The process might be performed in various electrolytes, including SBF (Wang et al. 2004; Lopez-Heredia et al. 2007). A typical example of the deposited coating is shown in Figure 6 (Layrolle 2011). A coating thickness of less than 1 μm can be achieved. Reduction of the thickness leads to an increased resistance to delamination, which is observed frequently for thicker coatings (Peng et al. 2006). Electrochemical deposition of nano-sized crystals is also possible (Shirkhanzadeh 1998; Yousefpour et al. 2006; Narayanan et al. 2007, 2008a, 2008b, 2008c. Natural materials, such as shells, have been tested as the source of calcium to produce coatings by electrochemical deposition (Narayanan et al. 2006). Unfortunately, deposition of calcium orthophosphates requires a sufficient volume of electrolyte to surface ratio. Besides, approximately 20 ml of electrolyte is needed to coat 1 cm^2^ of implant. Additionally, hydrogen gas production hampers the deposition due to formation of bubbles on the titanium surface, which results in non-uniform coatings (Layrolle 2011). In order to overcome the latter problem, a modulated electrochemical deposition technique has been proposed (Lin et al. 2003).

According to the literature, nucleation of calcium orthophosphate crystals during the electrochemical deposition can occur either as instantaneous nucleation or as progressive nucleation (Eliaz and Eliyahu 2007). Nucleation is said to be instantaneous whenever the formation rate of a nucleus at a given site is expected to be at least 60 times greater than the expected rate of coverage of the site by growth only. Nucleation is said to be progressive when the expected coverage of a site by growth is at least 20 times greater than the coverage of the same site by the act of nucleation. After being formed, calcium orthophosphate nuclei can grow in one, two or three dimensions resulting in different shapes of the deposits like needles, disks or hemispheres depending on deposit/substrate binding energy and their crystallographic misfit. In the electrochemical deposition of HA from aqueous electrolytes, during the first approximately 12 min, the nucleation is instantaneous and is accompanied by a two-dimensional growth. Subsequently, the nucleation becomes progressive and is accompanied by a three-dimensional growth (Eliaz and Eliyahu 2007).

In general, calcium orthophosphate coatings, layers or films obtained by the electrochemical method have a uniform structure since they are formed gradually through a nucleation and growth process at relatively low temperatures (de Groot et al. 1998). Such coatings might be porous (Duan et al. 2003). Interestingly, that in order to produce apatite coatings, non-apatitic calcium orthophosphates might be electrochemically deposited, followed by additional treatments (Redepenning et al. 1996; Han et al. 1999b, 2001; Kumar et al. 1999; Silva et al. 2001). Subsequently, the deposited calcium orthophosphate coatings, layers or films might be heat treated in water steam at 125°C (Shirkhanzadeh 1993) and/or then calcined at temperatures up to 800°C to densify and improve its bonding to the substrates.

##### Sol-gel deposition

By definition, a sol is a two-phase suspension of colloidal particles in a liquid, while gels are regarded as composites because they consist of a solid skeleton or network that encloses a liquid phase or an excess of the solvent. Therefore, the sol-gel process, as the name implies, is a wet-chemical technique that involves transition from a liquid ‘sol’ into a solid ‘gel’ phase. Colloidal particles can be in the approximate size range of 1 to 1,000 nm; hence, gravitational forces on these particles are negligible, and interactions are dominated by both short-range forces and surface charges. To prepare sols, usually, inorganic metal salts and/or organometallic compounds such as metal alkoxides are used as precursors. Sols are formed after a series of hydrolysis and condensation reactions of the precursors. Then, the sol particles condense into a continuous liquid gel phase. Besides, a sol might be prepared by dispersion of colloidal particles in a liquid, followed by destabilization of the sol to produce a particulate gel. With further drying and heat treatment, the gel is then converted into dense ceramic or glass materials (Morris 2011). The deposited gels create coatings, films and layers. Sol-gel coatings, films and layers are usually produced using spin or dip (Liu et al. 2002a) coating techniques (see below).

According to this technique, calcium orthophosphate coatings, layers or films are prepared by dipping the sample in calcium (usually, nitrate salt) and phosphorus (usually, alkyl phosphates) gels for an appropriate time at low reaction temperatures. As-formed coatings, layers or films are porous, less dense and have poor adhesion to the substrate. To improve their properties, the samples are annealed at temperatures of 400°C to 1,000°C (Gross et al. 1998c; Haddow et al. 1999; Montenero et al. 2000; Tkalcec et al. 2001; Liu et al. 2002b; Metikoš-Huković et al. 2003; Kim et al. 2004a; Gan and Pilliar 2004; Zhang et al. 2006; Stoica et al. 2008). Depending upon the temperature, different calcium orthophosphate compounds are obtained. The resulting coatings, layers or films can be extremely dense and adhere strongly to the underlying substrate (de Groot et al. 1998). Occasionally, in order to improve the bond strength between the coating and the substrate, an intervening layer of another compound might be applied prior the sol-gel deposition of a calcium orthophosphate (Kim et al. 2004b).

##### Biomimetic deposition

**Figure 7:**
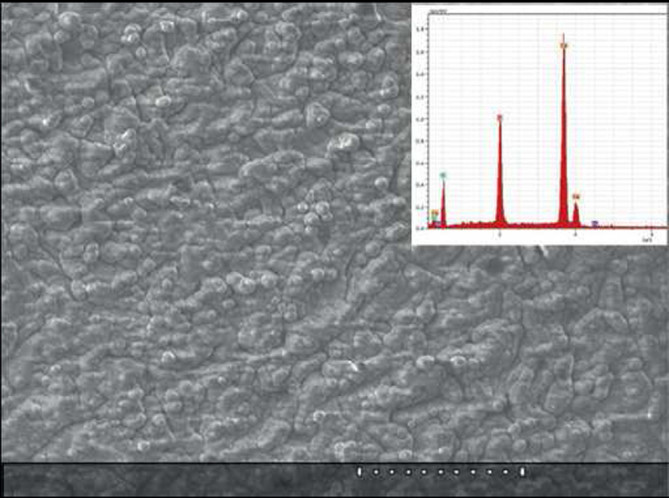
**A scanning electron microscopy of a typical biomimetically deposited carbonated apatite coating.** Inset: an EDX spectrum of the coating. Bar is 200 μm. Reprinted from Layrolle (2011) with permission.

Since biomimetics (synonyms: bionics, biomimicry) seeks to apply biological methods and systems found in nature, biomimetic deposition appears to be a method whereby a biologically active bone-like apatite layer is formed on a substrate surface by immersion in various simulating solutions, such as Hank's balanced salt solution (HBSS) or simulated body fluid (SBF) (Song et al. 2004; Habibovic et al. 2002; Oliveira et al. 2003; Hanawa and Ota 1992; Li et al. 1992; Leitão et al. 1995; Oliveira et al. 1999; Wang et al. 2003). This method involves a heterogeneous nucleation and growth of bone-like calcium orthophosphate crystals on the surface of implants at physiological conditions (temperatures 25°C or 37°C and solution pH within 6 to 8) for several days or even weeks. However, since all simulating solutions, such as HBSS and SBF (their chemical composition might be found in literature) contain a number of various ions, ion-substituted calcium orthophosphates might be deposited only. The thickness of such calcium orthophosphate coatings, layers or films varies within several microns (Table 3), while, according to the X-ray diffraction measurements, the majority of the biomimetic precipitates appear to be either amorphous or poorly crystalline (de Groot et al. 1998). A typical example of a biomimetically deposited calcium orthophosphate coating is shown in Figure 7 (Layrolle 2011).

The mechanism of bone-like apatite formation on an oxidized surface of titanium was investigated in details (Takadama et al. 2001; Uchida et al. 2003). Briefly, it looks as follows: First, a layer of amorphous sodium titanate is formed on the Ti surface after alkali pretreatment. Then, immediately after immersion into SBF, the sodium titanate exchanged Na^+^ ions for H_3_O^+^ ions in the fluid to form Ti-OH groups on its surface. Later, the Ti-OH groups incorporated calcium ions from the SBF to form a layer of amorphous calcium titanate. After longer soaking times, the amorphous calcium titanate incorporated orthophosphate ions from the SBF to form ACP coatings with a Ca/P atomic ratio of approximately 1.4. Thereafter, ACP converted into bone-like ion-substituted CDHA with a Ca/P ratio of approximately 1.65, which was close to the value of bone mineral (Takadama et al. 2001). In the next study, the authors specified that, after exchanging Na^+^ ions for H_3_O^+^ ions, various types of titania gels might be formed but only those with the anatase or rutile structure induced apatite formation (Uchida et al. 2003). Further specific details on this topic are available in literature (Kokubo and Yamaguchi 2011).

Since biomimetic deposition of calcium orthophosphate coatings, layers or films is a slow process, ways were sought to make it faster. Using condensed versions of the simulating solutions is the most popular option. For example, time for apatite induction in the 1.5-fold SBF was significantly shortened compared to that in the standard SBF. Therefore, the concentration of SBF was increased further, namely, 2-fold (Sun and Wang 2010; Miyaji et al. 1999; Kim et al. 2000), 5-fold (Barrere et al. 2002a, 2002b, 2004) and even 10-fold (Tas and Bhaduri 2004) SBF solutions were used to accelerate precipitation and increase the amount of precipitates. However, whenever possible, this should be avoided because the application of condensed solutions of SBF leads to changes in the chemical composition of the precipitates; namely, the concentration of carbonates increases, while the concentration of orthophosphates decreases (Dorozhkina and Dorozhkin 2003).

The nucleation and growth of calcium orthophosphate coatings deposited on Ti6Al4V substrates from 5-fold SBF were investigated in details by both atomic force and environmental scanning electron microscopes (Barrere et al. 2004). Scattered calcium orthophosphate deposits of approximately 15 nm in diameter were found to appear after only 10 min of immersion in 5-fold SBF. Then, they grew up to 60 to 100 nm after approximately 4 h. With increasing immersion time, the packing of calcium orthophosphate deposits with size of tens of nanometers in diameter formed larger globules and then continuous calcium orthophosphate coatings on Ti6Al4V substrates. The coatings were composed of nano-sized deposits. A direct contact between calcium orthophosphates and the Ti6Al4V surface was observed (Barrere et al. 2004). A stable solution containing high concentrations of calcium and orthophosphate ions was prepared in another study (Li et al. 2002b). This solution became supersaturated after NaHCO_3_ was added. A uniform coating of approximately 40-μm thickness was obtained on the substrate after immersion for 24 h. The coatings contained adjustable composition from CDHA to DCPD (Li et al. 2002b).

Simplification of the ionic composition of the standard simulating solutions is still another option to increase deposition kinetics (Bigi et al. 2005a; Li 2003). For example, a fast (a few hours instead of 14 days with SBF) biomimetic deposition of CDHA coatings on Ti6Al4V substrates was obtained using a slightly supersaturated Ca/P solution with an ionic composition simpler than that of SBF. Thin film XRD indicated that the deposits obtained after approximately 3 h were poorly crystalline CDHA, and their content increased on increasing the soaking time up to 3 days (Bigi et al. 2005a). However, since adhesion of this coating to the substrate was not indicated, it is doubtful whether this coating had sufficient strength to resist dissolution inside the body.

##### Dip coating

Dip coating is a popular way of creating coatings, films and layers for various purposes. It consists of several successive steps. A substrate is immersed into either a solution or a suspension of the coating material (in our case, calcium orthophosphate) at a constant speed. A wet coating, film or layer is deposited by itself on the substrate while it is pulled up. Usually, withdrawing is carried out at a constant speed to avoid any jitters. The speed determines the thickness (the faster, the thinner). Excess solution or suspension is drained from the surface. A solvent evaporates from the solution or suspension, forming a denser coating, film or layer. For volatile solvents, such as alcohols, evaporation starts already during the deposition and drainage steps. After being dried and sintered, a solid surface is achieved (Brinker et al. 1991). By means of dipping, uniform coatings, films and layers of calcium orthophosphates have been applied onto various substrates (Li et al. 1996; Weng and Baptisa 1998; Jiang and Shi 1998; Choi et al. 2003; Bini et al. 2009).

There are two mechanisms which govern the formation of the surface coatings, films or layers during dip coating. The first mechanism is known as liquid entrainment. It occurs when a specimen is withdrawn from slurry faster than it can drain from the surface, leaving a thin film (Pontin et al. 2005). The second mechanism is slip casting, in which the capillary suction caused by a substrate drives ceramic particles to concentrate at the substrate-suspension boundary, and a wet layer is formed (Gu and Meng 1999). The withdrawal velocity and the suspension properties (volume fraction of solids, viscosity) have influence on the liquid entrainment mechanism, while the surface microstructure of the substrate (porosity and pore diameter) together with the suspension properties have influence on the slip casting mechanism. By modifying these parameters, layers as thin as 2 μm and as thick as 0.5 mm might be formed (Pontin et al. 2005; Gu and Meng 1999).

##### Physical vapor deposition techniques

In general, all types of physical vapor deposition (or sputtering) techniques for producing coatings, films and layers can be broadly classified into two main groups: (1) those involving thermal evaporation techniques, where a material is heated until its vapor pressure becomes greater than the ambient pressure, and (2) those involving ionic sputtering methods, where a highly energetic beam of ions and/or electrons strikes a solid target and knocks atoms off from the surface (Narayanan et al. 2010; Paital and Dahotre 2009a). Usually, physical vapor deposition occurs in vacuum; however, it might be performed in presence of some gasses. The target is the source material (in our case, a calcium orthophosphate). Substrates are placed into a chamber, and they are pumped down to a prescribed pressure. Sputtering is driven by momentum exchange between the ions and atoms in the materials due to collisions. Afterwards, the dislodged atoms or molecules are deposited on a substrate which is also placed into the same vacuum chamber. An important advantage of the sputter deposition is that even materials with very high melting points are easily sputtered. For the efficient momentum transfer, the atomic weight of the sputtering gas should be close to the atomic weight of the target, so neon or argon is preferable for sputtering of light elements, while krypton or xenon is used for heavy elements (Cuerno and Barabási 1995). However, for deposition of calcium orthophosphates, oxygen might be used as well. It has a number of features, and a better stoichiometry with respect to HA of the deposited coatings, films and layers is one of them (van Dijk et al. 1997).

To sputter calcium orthophosphates, several types of the physical vapor deposition techniques are used, such as ion beam (Stevenson et al. 1989; Barthell et al. 1989; Ong et al. 1991, 1992, 1994; Yoshinari et al. 1994; Cui et al. 1997; Kim et al. 1998; Luo et al. 1999; Choi et al. 2000; Wang et al. 2001; Hamdi and Ide-Ektessabi 2003; Lee et al. 2003; Fujihara et al. 2004; Lee et al. 2005b; Rabiei et al. 2006; Lee et al. 2007a; Blalock et al. 2007), radio-frequency (RF) magnetron (Cooley et al. 1992; Yamashita et al. 1994; Jansen et al. 1993; Wolke et al. 1994; van Dijk et al. 1995, 1996; Wolke et al. 1998, 2003; Nelea et al. 2003, 2004; Feddes et al. 2003a, 2003b; Ding 2003; Yamaguchi et al. 2006; Wan et al. 2007; Ozeki et al. 2007; Ueda et al. 2007; Snyders et al. 2008; Ievlev et al. 2008; Toque et al. 2009), pulsed laser (Nelea et al. 2000, 2002, 2004; Cotell et al. 1992, Cotell 1^993^; Torrisi and Setola 1993; Cotell 1993; Singh et al. 1994; Wang et al. 1997; Hontsu et al. 1997; Fernández-Pradas et al. 1998, 1999, 2001; Mayor et al. 1998; Arias et al. 1998; Craciun et al. 1999; Fernandez-Pradas et al. 1999; Zeng et al. 2000; Cleries et al. 2000a; Nelea et al. 2000; Zeng and Lacefield 2000; Fernandez-Pradas et al. 2001; Nelea et al. 2002; Socol et al. 2004; Kim et al. 2005a; Bigi et al. 2005b; Koch et al. 2007; Kim et al. 2007a; Paital and Dahotre 2008; Paital et al. 2009; Dinda et al. 2009; Tri and Chua 2009; Sygnatowicz and Tiwari 2009), diode, direct current and reactive sputtering or deposition (Massaro et al. 2001). The physical and aggregate states of the calcium orthophosphate source might influence the deposition kinetics. For example, the deposition rate of HA was found to be much higher in a solid plate target than in a powder lump target, owing to the difference of apparent density approximately 75% and approximately 18%, respectively (Wan et al. 2007).

Depending on the type of sputtering system and parameters used for the deposition, the structure and chemical composition of the deposited coatings, layers and films may be quite different from those of the initial material used for sputtering. For example, differences in Ca/P ratios between the initial calcium orthophosphates and that in the sputtered coatings were suggested to be attributed to the preferential sputtering of calcium, probably due to a possibility of orthophosphate ions being pumped away before they are deposited at the substrate (Zalm 1989). It was also suggested that orthophosphate ions might be weakly bound to the growing coatings, layers and films, and therefore, they are sputtered away by incoming ions or electrons (van Dijk et al. 1996). Nevertheless, all sputtering techniques have the advantage of depositing thin coatings, films and layers with strong adhesion and compact microstructure.

##### Ion beam assisted deposition

Ion beam assisted deposition (IBAD) is a vacuum technique in which ions of a material to be deposited (in our case, calcium orthophosphates) are generated by collisions with electrons. Then, the detached ions are accelerated by an electric field emanating from a grid toward a target. As the ions leave the source, they are neutralized by electrons from the second external filament and form neutral atoms. A pressure gradient between the ion source and a sample chamber is generated by placing a gas inlet at the source and shooting through a tube into the sample chamber (Ali et al. 2010). Therefore, a typical deposition system consists of two main parts: electron or ion beam bombarding and vaporizing a calcium orthophosphate bulk target to produce an elemental cloud towards the surface of a substrate and a source for simultaneous irradiation of a substrate with highly energetic inert (e.g., Ar^+^) or reactive (e.g., O_2_^+^) gas ions to assist the deposition. Both single and dual ion beam assisted deposition systems are available. Good illustrations of both systems are presented in literature (Narayanan et al. 2010; Paital and Dahotre 2009a; Surmenev 2012).

**Figure 8:**
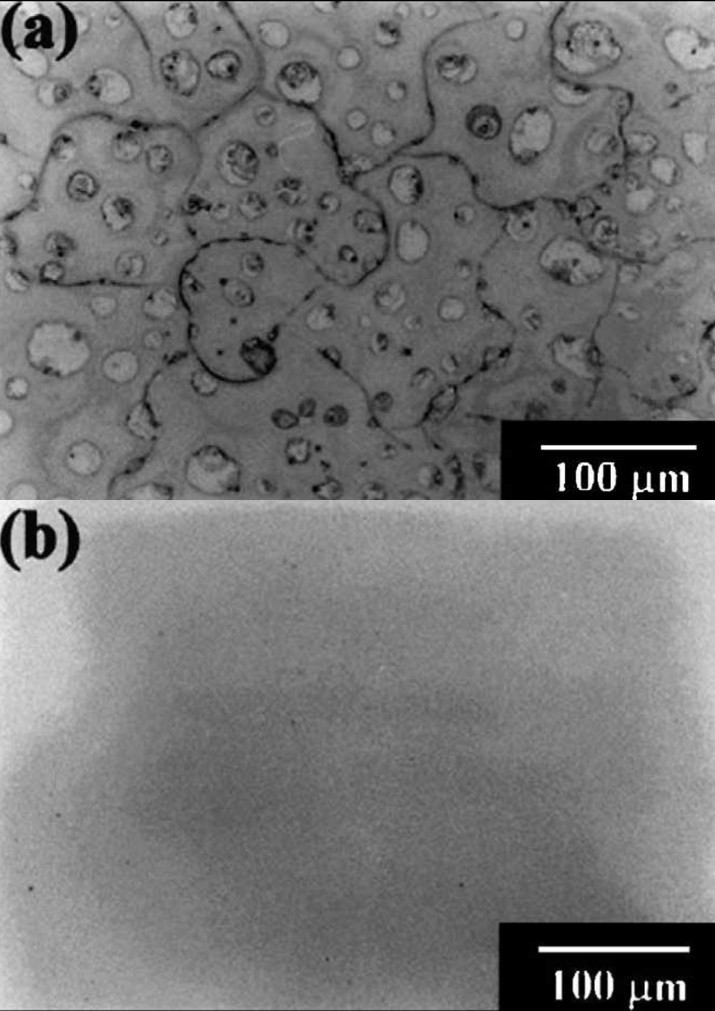
**Optical micrographs of a calcium orthophosphate layer deposited on a Si wafer.** (**a**) Without ion beam bombardments and (**b**) with Ar ion beam bombardments (120 V, 0.8 A). Reprinted from Lee et al. (2003) with permission.

In this approach, firstly thin (a few hundred atomic layers thick) and amorphous calcium orthophosphate coatings, layers or films are usually deposited. Then, an ion implantation technique, with ions such as argon, nitrogen and oxygen, is used to make them crystalline (Stevenson et al. 1989; Barthell et al. 1989; Ong et al. 1991, 1992, 1994; Yoshinari et al. 1994; Cui et al. 1997; Kim et al. 1998; Luo et al. 1999; Choi et al. 2000; Wang et al. 2001; Hamdi and Ide-Ektessabi 2003; Lee et al. 2003, 2007a; Fujihara et al. 2004; Lee et al. 2005b; Rabiei et al. 2006; Blalock et al. 2007). A high bond strength associated with this deposition technique appears to be a consequence of an atomic intermixing interfacial layer, which can be up to a few microns thick. Studies revealed alterations in the chemical composition of the ion beam deposited coatings, layers or films. For example, calcium orthophosphate films were synthesized on silicon wafers by electron beam evaporation of β-TCP both with and without simultaneous Ar ion beam bombardments (Lee et al. 2003). It was observed that a simultaneous bombardment with Ar ion beam had a significant effect on both the morphology (Figure 8) and composition of the films, namely, films formed without Ar ion beam bombardment were found to have a Ca/P ratio of approximately 0.76 and reacted immediately with the moisture in the air as soon as it is removed from the chamber. In contrast, the films formed with Ar ion beam bombardment had a Ca/P ratio of approximately 0.80 with smooth and featureless surface morphology (Lee et al. 2003).

**Figure 9:**
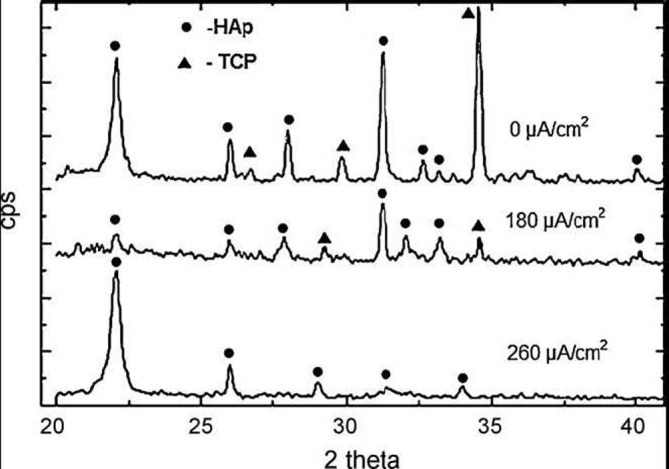
**XRD patterns of fully crystallized (after a heattreatmentat 1200°C) calcium orthophosphate coatings.** Sputtered at three different values of ion beam current density. Reprinted from Hamdi and Ide-Ektessabi (2003) with permission. HAp, hydroxyapatite; TCP, tricalcium phosphate.

In another study, calcium orthophosphate layers on silicon substrates were prepared by using ion beam assisted simultaneous vapor deposition. The method comprised of an electron beam heater and a resistance heater vaporizing CaO and P_2_O_5_, respectively, while an argon ion beam was focused onto substrates to assist the deposition (Hamdi and Ide-Ektessabi 2003). All deposited layers appeared to be amorphous, regardless of the current density level of the ion beam. Therefore, a post-heat treatment was applied to crystallize the layers. The effects of ion beam current density on the phase composition of the crystallized calcium orthophosphates are shown in Figure 9. The Ca/P ratio was found to increase with increasing ion beam current density presumably due to the high sputtering rate of P_2_O_5_ compared to that of CaO from the layer being coated. As seen in Figure 9, biphasic (HA + TCP) formulations were found when the ion beam was either not used or used at current density of 180 mA/cm^2^, while at ion beam current density of 260 mA/cm^2^, only HA peaks were observed (Hamdi and Ide-Ektessabi 2003). In still another study, the X-ray photoelectron spectroscopy analysis of the deposited calcium orthophosphate coatings on titanium revealed several distinct zones: (i) the ambient-exposed surface exhibited elevated concentrations of carbon due to atmospheric contamination; (ii) the bulk zone contained relatively constant concentrations of calcium, oxygen, phosphorus and fluorine, indicating the chemistry for calcium fluoride and FA formation; (iii) while the underlaying zone exhibited elevated titanium and oxygen photoelectron peaks, suggesting the coexistence of calcium orthophosphates within titanium oxides. Furthermore, the substrate was shown to be identical to the passivated titanium surface prior to deposition (Ong et al. 1991). A similar zone structure was also discovered by other researchers (Wang et al. 2001). In addition, a cross-section of functionally graded thin HA coatings on silicon substrate obtained by a dual ion beam assisted deposition and simultaneous heat treatment was investigated and the microstructural analysis of the coatings revealed a gradual decrease of the grain size and crystallinity towards the surface, leading to nano-scale grains and eventually amorphous layer at the surface (Rabiei et al. 2006).

Choi et al. (2000) deposited HA films on Ti-6Al-4 V alloy by electron beam vaporization of pure HA target and simultaneous bombardment using a focused Ar ion beam on the metal substrate to assist deposition. The effect of Ar ion beam current on the bond strength and dissolution of the coating in a physiological solution was studied. The bond strength between the coating and the substrate increased with increasing current, whereas the dissolution rate in physiological solution decreased remarkably (Choi et al. 2000). Further details on this technique are available in the aforementioned references.

##### Pulsed laser deposition or laser ablation deposition

Shortly after the discovery of a laser in the end of 1950s (Amy and Storb 1955), researchers began focusing their beams at materials to observe the interaction. PLD or laser ablation deposition technique for producing thin films became increasingly popular in 1970s due to the advent of lasers delivering nanosecond pulses (Singh and Narayan 1990). In this technique, a high power pulsed laser beam is focused inside a vacuum chamber to strike a target of the material (in our case, calcium orthophosphates) resulting in a gaseous cloud of various atoms, ions, molecules, molecular clusters and, in some cases, droplets and target fragments, due to a thermal decomposition of the target (Dinda et al. 2009). For sufficiently high laser energy density, each laser pulse vaporizes or ablates a small amount of the material, creating a plasma plume. The ablated material is ejected from the target in a highly forward-directed plume. The ablation plume provides the material flux, which then is deposited on a substrate. This process can occur in both ultra-high vacuum and presence of a background gas, such as oxygen, which is commonly used when depositing oxides to fully oxygenate the deposits. Argon (Bao et al. 2006) and water vapor (Fernandez-Pradas et al. 2000) can be used as well. The thorough investigation of a plasma plume expansion process during an ArF laser ablation of HA is well described elsewhere (Jedynski et al. 2008). The experimental setup of a PLD technique is available in literature (Narayanan et al. 2010; Paital and Dahotre 2009a; Surmenev 2012); it essentially consists of a laser source, an ultrahigh vacuum deposition chamber equipped with a rotating target and a fixed substrate holder plus pumping systems. Mostly, the substrates are attached to the surface parallel to the target surface at a target-to-substrate distance of 2 to 10 cm. Usually, for ablation, ultraviolet excimer lasers with pulses of approximately 10-ns duration and power densities in the order of 10 to 500 MW/cm^2^ are required. A two-laser beam technique (so-called, laser-assisted laser ablation method) is used as well (Katayama et al. 2009). In this technique, one laser beam from KrF laser, the ablation laser, is used for ablation of a HA target. The other beam from ArF laser, the assist laser, is used to irradiate a Ti substrate surface during formation of the HA coating. The assist laser plays an important role in the formation of a crystalline HA coating and improves the strength of adhesion to the Ti substrate (Katayama et al. 2009). Further details on the PLD technique might be found in literature (Willmott and Huber 2000).

A PLD process is used for forming thin (0.05 to 5 μm) calcium orthophosphate coatings, layers or films on various substrates (Nelea et al. 2000, 2002, 2004; Cotell et al. 1992, 1993; Torrisi and Setola 1993; Singh et al. 1994; Wang et al. 1997; Hontsu et al. 1997; Fernández-Pradas et al. 1998, 1999, 2001; Mayor et al. 1998; Arias et al. 1998; Craciun et al. 1999; Fernandez-Pradas et al. 1999; Zeng et al. 2000; Cleries et al. 2000a; Zeng and Lacefield 2000; Socol et al. 2004; Kim et al. 2005a, 2007a; Bigi et al. 2005b; Koch et al. 2007; Paital and Dahotre 2008; Paital et al. 2009; Dinda et al. 2009; Tri and Chua 2009; Sygnatowicz and Tiwari 2009). The process involves ablation of a calcium orthophosphate (usually, HA) target using a pulsed (usually, pulses of 30 ns and 120 mJ at a repetition of 10 Hz) KrF excimer laser beam (*λ* = 248 nm) in 0.3 Torr/H_2_O atmosphere and deposition of the ejected HA material on a heated (400°C to 800°C) substrate. The deposition rate of PLD is about 0.02 to 0.05 nm per laser shot (de Groot et al. 1998). An investigation into the effects of high laser fluence (between 2.4 J/cm^2^ and 29.2 J/cm^2^) on the properties of calcium orthophosphate films was performed (Tri and Chua 2009). The films deposited at 2.4 J/cm^2^ were found to be partially amorphous and had rough surfaces with many droplets, while higher laser fluences showed a higher level of crytallinity and lower surface roughness. Furthermore, higher laser fluences also decreased the ratio Ca/P of as-deposited films and, probably, increased their density (Tri and Chua 2009). The substrate heating is necessary to ensure the formation of a highly crystalline and phase pure coatings, films and layers. Besides, the substrate temperature could be varied to provide deposits with the desired fine texture and roughness, depending on their application (Saju et al. 2009; Rau et al. 2010).

Typically, during the deposition, a target should be rotated to achieve a stable ablation rate. As PLD is usually carried out at high substrate temperatures, a thin oxide layer might be formed on the substrate surface prior to the deposition of calcium orthophosphates and, thereby, it influences its adherence to the substrate (Nelea et al. 2000). The deposited coatings, layers and films frequently consist of several calcium orthophosphates (often with admixtures of other substances, such as CaO, calcium pyrophosphates, etc.) and might contain both amorphous and crystalline phases (Cleries et al. 2000a; Koch et al. 1990). Biphasic formulations, such as HA + TTCP (Kim et al. 2005b), might be deposited as well. Interestingly, TTCP in the coatings was not formed by partial conversion of previously deposited HA. Instead, it was produced by nucleation and growth of TTCP itself from the ablation products of the HA target or by accretion of TTCP grains formed during ablation (Kim et al. 2005b). Furthermore, the PLD-deposited coatings, films and layers might consist of calcium orthophosphates with different morphologies (e.g., granular and columnar), which have different resistance values to delamination (Cleries et al. 2000a). More to the point, various types of oriented textures might be created as well (Kim et al. 2005a, 2007a, 2010). A modification known as ‘transmission laser coating’ has been introduced (Cheng and Ye 2010).

Further details on the PLD technique might be found in literature (Surmenev 2012; Koch et al. 2007).

##### Magnetron sputtering

A magnetron is a high-powered vacuum tube that generates microwaves using the interactions of a stream of electrons with a magnetic field. Magnetron sputtering technique has been emerged in the mid of 1960s (Gill and Kay 1965) and is considered as a high-rate vacuum coating technique for depositing metals, alloys and compounds onto a wide range of materials with thickness up to approximately 5 μm (Table 3). A sputtering system consists of an evacuated chamber, a wave generator, a magnetron, a cooling system, as well as it contains a target and a substrate. It works on the principle of applying a specially shaped magnetic field to a sputtering target. Once the substrate is placed into the vacuum chamber, air is removed, and the target material (in our case, calcium orthophosphates) is released into the chamber in the form of a gas. Powerful magnets ionize particles of the target material. Then, the negatively charged target material lines up on the substrate to form deposits (Kelly and Arnell 2000). In principle, magnetron sputtering can be done in either DC (direct current) or RF modes; however, since the DC mode might be done with conducting materials only, the RF mode is solely used to deposit calcium orthophosphates. Typically, RF magnetron sputtering employs a sinusoidal wave generator operating at 13.56, 5.28 or 1.78 MHz. The parameters that directly affect the quality and integrity of calcium orthophosphate coatings, films and layers include discharge power, gas flow rate, working pressure, substrate temperature, deposition time, post-heat treatment and negative substrate bias (Surmenev 2012). For example, the deposition rate was found to increase with increasing argon gas pressure up to 2 Pa but decreased significantly as the pressure increased up to 5 Pa, while the Ca/P ratios of as-deposited coatings decreased significantly at the higher argon gas pressures (Boyd et al. 2007). A good schematic setup of a magnetron sputtering system is available in literature (Narayanan et al. 2010; Paital and Dahotre 2009a; Surmenev 2012).

For the first time, RF magnetron sputtering was used to prepare calcium orthophosphate coatings in 1992 (Cooley et al. 1992). Since then, it has become a convenient method for deposition of biocompatible ceramic coatings, layers and films on various substrates (Yamashita et al. 1994; Jansen et al. 1993; Wolke et al. 1994, 1998, 2003; van Dijk et al. 1995, 1996; Nelea et al. 2003, 2004; Feddes et al. 2003a, 2003b; Ding 2003; Yamaguchi et al. 2006; Wan et al. 2007; Ozeki et al. 2007; Ueda et al. 2007; Snyders et al. 2008; Ievlev et al. 2008; Toque et al. 2009). The advantages of magnetron sputtering over other sputtering processes include a high deposition rate, an excellent adhesiveness and an ability to coat implants with difficult surface geometries (Table 3). Still, several issues, such as the endurance and the Ca/P ratio, have to be solved before magnetron sputtering can be applied to deposit, on a routine basis, pure and crystalline calcium orthophosphates on implant surfaces. For example, both microstructure and mechanical properties of HA thin films, grown on Ti-5Al-2.5Fe alloys by RF magnetron sputtering, were investigated (Nelea et al. 2003). The deposition was performed from pure HA target in low pressure Ar or Ar-O_2_ mixtures at substrate temperatures ranging from 70°C to 550°C. Smooth (an average roughness of approximately 50 nm) and uniform calcium orthophosphate films were fabricated. It was observed that the films grown at the substrate temperatures below approximately 300°C were prevalently amorphous (ACP) and contained a small amount of crystalline phases. On the contrary, the films obtained at a substrate temperature of 550°C or the films grown at room temperature followed by annealing at 550°C consisted of HA (Nelea et al. 2003).

The chemical composition of the deposited calcium orthophosphate coatings, films and layers might be modified by varying the RF sputtering power density (Snyders et al. 2008), namely, when the power density was increased by 240%, the Ca/P ratio increased from approximately 1.51 to approximately 1.82. X-ray diffraction indicated the phase pure HA except for the samples prepared at the highest power density values, in which the presence of CaO and TCP was also detected. Interestingly, deviations from the stoichiometric HA resulted in reduction of the elastic modulus, namely, for Ca/P approximately 1.51, the elastic modulus dropped by approximately 15%, which was attributed to Ca vacancies in the lattice, while for Ca/P approximately 1.82, the average elastic modulus decreases by approximately 10% due to formation of additional phases (Snyders et al. 2008).

Various types of calcium phosphates were magnetron sputtered from TTCP, HA, β-TCP, β-calcium pyrophosphate (CPP) and β-calcium metaphosphate (CMP) powder targets (Ozeki et al. 2007). The composition of the deposited films was changed depending on the target materials, while the Ca/P molar ratios of the films varied from 0.74 to 2.54, increasing with the Ca/P molar ratio of the target. Interestingly, the deposition rate of the aforementioned calcium phosphates was established as the following: TTCP ≈ β-CMP > β-TCP > β-CPP ≈ HA, which correlated well to the solubility order: TTCP ≈ β-CMP > β-TCP > β-CPP ≈ HA (Ozeki et al. 2007).

RF magnetron sputtering might be combined with other deposition techniques. For example, plasma-assisted RF magnetron co-sputtering deposition method was used to deposit calcium orthophosphates on Ti6Al4V orthopedic alloy (Xu et al. 2005; Long et al. 2007). Further details on the magnetron sputtering technique are available in excellent reviews (Surmenev 2012; Shi et al. 2008).

#### Other deposition techniques: miscellaneous

Prior describing the below mentioned deposition techniques, one should note that they are rare and are mentioned in just a few research papers. Therefore, the detailed description is not always possible.

##### Hot isostatic pressing

Hot isostatic pressing (HIP) is a manufacturing process used to reduce porosity and increase the density of many types of materials. The HIP process subjects a component to both elevated temperature and isostatic gas pressure in a high-pressure containment vessel. To deposit coatings, initially, solid cores are covered by a calcium orthophosphate (usually HA) powder. Both organic binders and some other additives are used to simplify deposition. A furnace is constructed within the high-pressure vessel, and the coated samples are placed inside to be pressed. Then, the specimens are heated at temperatures within 700°C to 1,200°C and pressed at pressures within 20 to 100 MPa. The obtained coatings, films and layers are usually thick (0.2 to 2.0 mm) and dense (Bocanegra-Bernal 2004).

The HIP technique was used to manufacture calcium orthophosphate coatings, films and layers on various materials (Lacefield 1988; Herø et al. 1994; Wie et al. 1998; Kameyama 1999). For example, HA granules (32 to 38 μm in diameter) were implanted into a substrate of superplastic titanium alloy. First, the HA granules were spread over this surface and, then, hot pressed at 750°C and 17 MPa for 1 h with a plunger to implant them into the substrate. After 10 min of implantation, the implantation ratio was approximately 20%, and some granules were not on the substrate. After 60 min of implantation, the implantation ratio was 100%, but the upper areas of granules were exposed (Kameyama 1999).

A variation in the HIP technique was proposed in which thin HA coatings were prepared with a curved surface at low temperatures (Onoki and Hashida 2006). The method used double-layered capsules in order to create suitable hydrothermal conditions; the inner capsule encapsulated the coating materials and a Ti substrate, while the outer capsule was subjected to isostatic pressing under the hydrothermal conditions. It was demonstrated that a HA layer of approximately 50 μm thick could be deposited on Ti cylindrical rods at 135°C under the confining pressure of 40 MPa. The deposited HA layer had a porous microstructure with the relative density of approximately 60%. According to the results of pullout tests, the shear strength was in the range of 4.0 to 5.5 MPa. These results also revealed that a crack propagation occurred within the HA coating layer but not along the HA/Ti interface. This observation suggests that the fracture property of the HA/Ti interface was higher than that of the HA ceramics only. Thus, hydrothermal HIP technique appears to be a useful method for producing bioactive HA ceramic coatings on curved prostheses surfaces (Onoki and Hashida 2006). However, the majority of the calcium orthophosphate coatings, layers or films produced by HIP technique are contaminated with metal and SiO_2_ particles due to the use of a glass encapsulating tubes (de Groot et al. 1998).

##### Frit enameling

Frit is a ceramic composition that was fused in a special fusing oven, quenched to form a glass and granulated. According to this technique, a metal bar was dipped into a HA slurry, dried and sintered at 1,100°C to 1,200°C for a minimum of 3 h in a protective Ar atmosphere (Lacefield 1988). Coatings created in this technique show very low interfacial shear strength (approximately 0.22 MPa). Probably, the furnace atmosphere used for sintering was inadequate, which resulted in excessive substrate oxide layer thickness and poor bonding (de Groot et al. 1998).

##### Aerosol-gel

An aerosol is a colloid suspension of fine solid particles or liquid droplets in a gas. Examples are clouds and air pollution, such as smog and smoke. While gels are regarded as composites because they consist of a solid skeleton or network that encloses a liquid phase or an excess of the solvent. Therefore, the aerosol-gel process, as the name implies, is a gas-chemical technique that involves transition from a gaseous ‘aerosol’ into a solid ‘gel’ phase.

The aerosol-gel technique was also applied to produce highly porous calcium orthophosphate coatings on various materials (Manso et al. 2002a; 2002b; Manso-Silván et al. 2003). Calcium nitrate and triethylphosphate diluted in ethanol were used as precursor solutions. After production of a steady state aerosol, the microdroplets were conducted into a deposition chamber by an air flux. After being deposited, the coatings were sintered at temperatures within 500°C to 1,000°C. The composition, structure and morphology of the final coatings were found to fit highly porous polycrystalline HA. The adhesive strength, measured by means of indentation techniques, was found to be in the order of 100 MPa, which was significantly higher than the values obtained by sol-gel deposition technique (Manso et al. 2002a; 2002b; Manso-Silván et al. 2003).

##### Micro-arc oxidation

Micro-arc oxidation (MAO), also called plasma electrolytic oxidation, anodic spark deposition, or micro-arc discharge oxidation, is a plasma-chemical and electrochemical process. The process combines electrochemical oxidation with a high-voltage spark treatment in an aqueous electrolytic bath, which also contains modifying elements in the form of dissolved salts (*e.g.*, silicates) to be incorporated into the resulting coatings. A schematic setup of a MAO system is available in literature (Narayanan et al. 2010; Paital and Dahotre 2009a).

By means of a MAO technique, calcium orthophosphate coatings, films and layers have been prepared on various metals (Song et al. 2004; Liu et al. 2005; Sun et al. 2007; Wei et al. 2007; Han et al. 2008). For example, MAO was performed on titanium in an electrolyte containing calcium glycerophosphate and calcium acetate using a direct current power supply. The MAO technique appeared to be suitable to form porous and rough ceramic coatings containing Ca and P. Then, the coatings were hydrothermally treated in aqueous solutions with pH within 7.0 to 11.0 (adjusted by adding NaOH) at 190°C for 10 h in an autoclave. This procedure converted undisclosed calcium orthophosphates into CDHA and/or HA crystals, while the amount of precipitated HA increased with solution pH increasing (Liu et al. 2005).

##### Direct laser melting

According to the direct laser melting technique, a starting precursor (calcium orthophosphate) powder is mixed thoroughly in a water-based organic solvent. Then, the suspension is sprayed onto the substrate surfaces to create a coating, which is then air dried to remove the moisture, followed by direct laser melting using either continuous wave or pulsed laser beams to produce strong bonds between the calcium orthophosphate coating and the substrate (Paital and Dahotre 2007, 2008, 2009a, 2009b; Kurella and Dahotre 2006. A schematic setup of a direct laser melting system is available in literature (Paital and Dahotre 2009a).

##### Laser cladding

Calcium orthophosphate coatings, films and layers were synthesized on various substrates by laser cladding (Lusquiños et al. 2001, 2003, 2005). Cheap precursors, such as mixed powders of calcium carbonate and DCPA/DCPD, can be used to prepare coatings (Wang et al. 2008b; Zheng et al. 2010; Lü et al. 2011a, 2011b; Lv et al. 2012). The reactions between CaCO_3_ and DCPA/DCPD can produce high crystallized HA in the coatings, as well as TTCP, α-TCP, β-TCP, Ca_2_P_2_O_7_ and CaO, if the Ca/P ratio is deviated from 1.67. Since the reactions between the powders produce gaseous by-products (CO_2_ and water vapor), the prepared coatings were porous. Furthermore, when deposition was performed on metals (e.g., Ti), other admixtures, such as CaTiO_3_, were formed. As the laser power increased, the amount of TTCP, HA and CaO in the coatings decreased gradually and, finally, only α-TCP and CaTiO_3_ remained. Nevertheless, the amount of HA could be increased greatly by heat treatment at 800°C for 5 h followed by furnace cooling, due to the total transformation of TTCP and α-TCP to HA (Wang et al. 2008b; Zheng et al. 2010; Lü et al. 2011a, 2011b; Lv et al. 2012).

##### Detonation gun spraying

Detonation gun spraying is a high temperature and a high velocity technique which is thought to introduce a higher degree of melting to starting powder. This technique has also been explored to prepare calcium orthophosphate coatings on titanium alloys (Gledhill et al. 1999, 2001). In this process, a mixture of oxygen and acetylene is fed into a barrel together with a charge of the power. The gas is ignited, and detonation waves accelerate the powder up to about 750 m/s. The process produces a denser coating, which has a higher proportion of the amorphous phase with some evidence for the appearance of β-TCP. A lower crystallinity and higher residual stress found in the detonation gun sprayed coatings resulted in a faster dissolution rate both *in vitro* and *in vivo* (Gledhill et al. 1999, 2001).

##### Cold spraying technique

In recent years, a new coating technology, known as cold spraying, has been developed (Gärtner et al. 2006). In this process, spraying particles (1 to 50 μm in size) experiencing both a little change in microstructure and a little oxidation or decomposition are accelerated by a supersonic jet of a compressed gas stream passing through a Laval type nozzle to a very high velocity (300 to 1,200 m/s). The deposition system consists of a gas pressure regulator, a gas heater, a powder feeder and a spray gun. In this technique, the deposited calcium orthophosphate particles are always in a solid state and at temperatures below their melting point. Thus, all phenomena occurring at high temperatures, such as thermal decomposition and phase transformations (see section Thermal spraying techniques above), are avoided. Deposition of calcium orthophosphate particles takes place through intensive plastic deformations (Zhang and Zhang 2011; Zhang et al. 2012a, 2012b). However, for successful bonding, the deposited particles have to exceed a critical velocity on impacts, which is dependent on the properties of the particular sprayed material (Gärtner et al. 2006).

##### Thermal substrate deposition

Thermal substrate deposition technique is based on the solubility differences at low and high temperatures, namely by heating a substrate in suitable saturated aqueous solutions, coatings, films and layers can be directly deposited onto the substrate. Various heating techniques of substrates have been proposed, namely conductive substrates, such as foil or wire, can be heated by electric current through them. Non-contact techniques, such as high frequency induction, can be used to heat materials with complex shapes. In either case, the immersed metallic sample can be heated up to 160°C in solutions, giving local supersaturations to perform crystallization.

Using this approach, calcium orthophosphate coatings, films and layers were obtained on titanium (Ziani-Cherif et al. 2002; Okido et al. 2002; Kuroda et al. 2002a, 2002b, 2003, 2005). An alternating current is passed through the metallic sample immersed in an aqueous solution containing calcium and phosphorus compounds. The deposition is usually performed for 10 to 30 min at solution pH 4 to 8. The type of precipitates varies, depending on the solution pH, temperature and ion concentrations, namely, precipitates of high quality, whose predominant component was HA (at pH > 6) or DCPA (at pH = 4), were obtained on titanium substrates by this technique. The content of HA in the deposits was found to increase with increasing temperature and heating time (Ziani-Cherif et al. 2002; Okido et al. 2002; Kuroda et al. 2002a, 2002b, 2003, 2005).

##### Matrix-assisted pulsed laser evaporation

Matrix assisted pulsed laser evaporation technique was developed as an alternative to PLD for delicate and accurate deposition of calcium orthophosphate films, coatings and layers combined with organic and/or biologic materials. The examples include deposition calcium orthophosphate-based biocomposites with sodium maleate (Negroiu et al. 2008), alendronate (Bigi et al. 2009) and silk fibroin (Miroiu et al. 2010). Thermally unstable calcium orthophosphates, such as OCP (Boanini et al. 2012), might be deposited as well. This technique provides a more gentle mechanism for transferring different compounds, including large molecular weight species, and it is expected to ensure an improved stoichiometric transfer, a more accurate thickness control and a higher uniformity of the coatings.

##### Electrostatic spray deposition

Electrostatic spray deposition (ESD) is based on generation of an aerosol composed of organic solvents containing inorganic precursors under the influence of high voltages. According to this technique, spray droplets are generated by pumping a solution through a nozzle. Between the nozzle and substrate, a high voltage is applied. Consequently, droplets coming out the nozzle are dispersed into a spray, and this spray is deposited upon the substrate. When the solvent has evaporated, a coating is formed. The schematic setup of the ESD technique is available in literature (Leeuwenburgh et al. 2003, 2004, 2005a).

**Figure 10:**
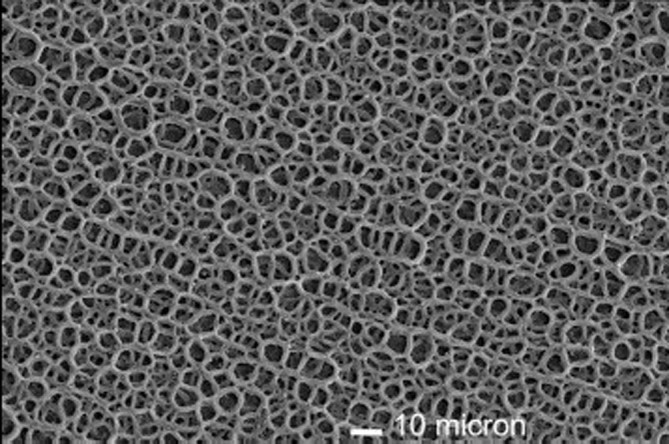
**A scanning electron microscopy of an electrostatic spray deposited calcium orthophosphate coating.** Characterized by a porous surface morphology. Reprinted from Leeuwenburgh et al. (2006a) with permission.

To perform ESD, a soluble calcium salt (nitrate or chloride) and phosphoric acid were dissolved in an alcohol. The obtained solutions were pumped, quickly mixed prior the nozzle and electrostatically sprayed onto a substrate, while the substrate itself might be heated to 300°C to 450°C (Leeuwenburgh et al. 2003, 2004, 2005a, 2005b, 2006a, 2006b). Besides, calcium orthophosphate powders might be suspended in alcohols, and the obtained suspensions are electrostatically sprayed (Lee et al. 2007b; Jiang et al. 2008; Iafisco et al. 2012). The chemical and morphological characteristics of the deposited calcium orthophosphate coatings, films and layers were found to be strongly dependent on both the composition of the precursor solutions (pH, absolute and relative precursor concentrations) and the deposition parameters, such as temperature, the nozzle-to-substrate distance, the liquid flow rate, as well as the geometry of the spraying nozzle. By varying these parameters, several phases and phase mixtures might be deposited by ESD technique: carbonate apatite, carbonated HA, α-TCP, β-TCP, DCPA, β- and γ-calcium pyrophosphates, calcium metaphosphate, CaCO_3_, CaO (Leeuwenburgh et al. 2005b, 2006a, 2006b). Since ESD might be performed at ambient temperatures, thermally unstable compounds could be deposited. As seen in Figure 10, the electrostatically sprayed calcium orthophosphate coatings, layers and films might be porous (Leeuwenburgh et al. 2006a, 2006b; Lee et al. 2007b; Jiang et al. 2008; Iafisco et al. 2012; Zhu et al. 2012). Nevertheless, after the deposition, the coated samples might be annealed at high temperatures. The annealing stage is necessary to aggregate and/or melt the deposited calcium orthophosphate particles and form highly dense and homogeneous coatings.

To conclude this part, combined techniques, such as sol-gel-assisted electrostatic spray deposition (Kim et al. 2007b) and electrostatic spray-assisted vapor deposition (Hou et al. 2007), have been developed as well. Further details on the ESD techniques are available in the aforementioned references.

##### Spin coating

Spin coating is a procedure used to apply uniform thin films to flat substrates. It is rather similar to dip coating. The coating process consists of four stages: deposition, spin up, spin off and evaporation. In this process, a sample is dipped in a solution or suspension and then withdrawn at a constant speed, usually with the help of a motor. Rotational draining and solvent evaporation result in the deposition of a coating, film or layer. A machine used for spin coating is called a spin coater, or simply spinner (Mennicke and Salditt 2002). Just a few publications on spin coating of calcium orthophosphates were published (You et al. 2005; Yuan et al. 2009; Carradò and Viart 2010).

### Properties

Generally, for clinical applications, slowly or non-resorbable high crystalline coatings, films and layers have been recommended in order to retain the bonding strength with implants. However, this contradicts to the statement that the ideal interface between the implants and surrounding tissues should match the tissues being replaced. For example, in the case of HA, its crystallinity has been stated to be in the inverse proportion to its bioactivity (LeGeros 2008). Therefore, from the bioactivity point of view, calcium orthophosphate coatings, films and layers should be of low crystallinity and also contain various ionic substitutions, such as sodium, magnesium and carbonate. Thus, since one of the first steps in bonding involves dissolution of the coating surface, it might be suggested that coatings, films and layers prepared from less crystalline and/or more resorbable calcium orthophosphates would be more beneficial for early bone ingrowth than those prepared from high crystalline HA (Narayanan et al. 2010). However, soluble coatings, films and layers will weaken the bonding strength between them and substrates. In particular, a rapid dissolution of coatings, films and layers may loosen the bonding strength between the implant surface and the host bone. For example, a comparative study on the biological stability and osteoconductivity of HA coatings on Ti produced by pulsed laser deposition and plasma spraying was conducted. After 24 weeks of implantation, the plasma sprayed HA coatings showed considerable instability and reduction in thickness but no statistical difference to the uncoated Ti (the control), while the pulsed laser deposited ones remained almost intact but showed a significantly higher amount of bone apposition (Peraire et al. 2006). Thus, in that study the coating stability prevailed over its solubility. Furthermore, the excessive amount of the dissolved ions from the soluble coatings, films and layers may cause local inflammatory reactions.

Except for crystallinity and chemical composition, a number of other factors appear to influence the physical, chemical and mechanical properties of calcium orthophosphate coatings, films and layers. They include thickness (this will influence adhesion and fixation - the agreed optimum now seems to be within 50 to 100 μm), phase and chemical purity, fatigue resistance, porosity and adhesion (de Groot et al. 1998; Sun et al. 2001). Abrasion resistance might be important as well (Morks et al. 2007).

#### Fatigue properties

Several studies have already demonstrated that cyclic loading of the coated samples leads to fatigue failure. Further, it has been shown that a combination of an aqueous environment with stress can result in delamination or accelerated dissolution of calcium orthophosphate coatings, which can influence the long-term stability of the implants (Kummer and Jaffe 1992; Reis et al. 1994; Wolke et al. 1997). For example, calcium orthophosphate coatings were RF magnetron sputtered on Ti-6Al-4 V bars and, afterwards, some of them were annealed at 650°C to convert ACP into crystalline structure. Then, the coated samples were mechanically tested in either dry or wet (SBF solution) conditions (Wolke et al. 1997). The results of SEM demonstrated that, after cyclic loading conditions in air, the bars coated by crystalline calcium orthophosphates showed a partial coating loss. Furthermore, in wet conditions only the heat-treated sputter-coated bars appeared to be stable. On the other hand, the ACP coatings showed signs of delamination in more stressed regions only (Wolke et al. 1997). Thus, the fatigue properties of amorphous and crystalline calcium orthophosphate coatings, films and layers are different. Furthermore, the fatigue behavior shows substantial differences when tested in either dry or wet/conditions.

#### Thickness

Depending on the deposition technique, the thickness of the calcium orthophosphate coatings, films and layers varies from nanometric dimensions to several millimeters (Table 3), and this parameter appears to be very important, namely, if calcium orthophosphate coatings, films and layers are too thick, they are easy to break. Furthermore, the outer layers might tend to detach from the inner ones. On the contrary, if calcium orthophosphate coatings, films and layers are too thin, they are easy to dissolve because resorbability of HA, which is the second least soluble among calcium orthophosphates (Table 1), is about 15 to 30 μm per year under the physiological conditions (Gineste et al. 1999). To complicate the situation, the failure mechanisms for thinner and thicker coatings, films and layers appear to be different, namely the failure mode of thinner (50 μm) HA coatings on a Ti alloy was found to be conclusively at or near the coating/bone interface, while that of thicker (200 μm) HA coatings was found to be at the coating/bone interface, inside the HA lamellar splat layer, as well as at the coating/Ti alloy substrate interface (Wang et al. 1993; Yang et al. 1997). A similar conclusion was made in another study, in which the mechanical behavior of thin (0.1, 1 and 4 μm) calcium orthophosphate coatings was compared (Vercaigne et al. 2000a). Considering these points, commercial plasma-sprayed HA coatings, films and layers have thicknesses between 50 and 200 μm (Sun et al. 2001), though cells and tissues interact with only top surface, and thus, thickness of approximately 10 nm would be sufficient for cell activity.

Calcium orthophosphate coatings of various thickness ranging from 170 nm up to 1.5 μm were obtained depending upon the deposition times (Fernandez-Pradas et al. 2001). The coating morphology was found to be grain-like particles and droplets. During growth, the grain-like particles grew in size, partially masking the droplets, and a columnar structure was developed. The thinnest (170 nm) coating consisted mainly of ACP. The coating of approximately 350-nm thick also contained HA, whereas even thicker coatings contained some α-TCP in addition to HA. All coatings failed under the scratch test by spalling from the diamond tip; however, the failure load increased as thickness decreased until only plastic deformation and cohesive failure for the thinnest coating was observed (Fernandez-Pradas et al. 2001). Therefore, both the structure and the phase composition of calcium orthophosphate coatings, films and layers might depend on their thickness.

#### Adhesion

In surgical practice, failure of implants and undesirable tissue responses take place when decohesion of coatings occurs. Therefore, all types of coatings, films and layers must adhere satisfactorily to the underlying substrate irrespective of their intended functions. Generally, the bottom surfaces of the coatings, films and layers are not in the full contact with the substrates. The areas that are in contact are called ‘welding points’ or ‘active zones’. Voids of various shapes and dimensions are located among them. In general, the greater the contact area, the better adhesion of the coating is (Pawlowski 2008). Since the chemical interactions between deposited calcium orthophosphates and substrates are rare, mechanical anchorage is the main mechanism involved in adhesion of calcium orthophosphate coatings, films and layers, in which the substrate surface roughness is the paramount parameter to achieve good adhesion. In many cases of ceramic coatings, the adhesion strength is found to be a linear function of the average surface roughness. Therefore, substrate preparation techniques, such as grit blasting, are used to increase roughness prior to spraying and, hence, increase the adhesion strength (see section ‘Brief knowledge on the important pre- and post-deposition procedures’). On the other hand, the amount of mechanical anchorage is reduced if a large amount of shrinkage occurs during solidification of the particles (Heimann 2006). Since the strength of human bones is approximately 18 MPa, all types of coatings, films and layers on the implant surface should have higher or, at least, comparable bond strength. Thus, according to the ISO requirements, the adhesion strength of calcium orthophosphate coatings, films and layers should not be less than 15 MPa (ISO 2000; ISO 2008).

Specifically, the adhesion forces of calcium orthophosphate coatings, films and layers should be high enough to maintain their bioactivity after a surgical implantation. Generally, tensile adhesion testing according to standards ASTM C633 (ASTM C633 2008) and ASTM F-1147-05 (2011) is the most common procedure to determine the quantitative adhesion values to the underlying substrates. Furthermore, fatigue (Surmenev 2012; Mukherjee et al. 2000), scratch (Cheng et al. 2009; Hamdi et al. 2010) and pullout (Cheng et al. 2009) testing, as well as wear resistance (Hamdi et al. 2010), are among the most valuable techniques to provide additional information on the mechanical behavior of calcium orthophosphate coatings, films and layers. Changes in the surface topography can give an indication of wear resistance. For example, coatings with good adherence to the substrate have shown less alteration of its surface roughness, while the study on the different parameters revealed that deposition time was the most influential factor in the wear behavior (Hamdi et al. 2010). The latter was attributed to its correlation with coating thickness. The scratch test is performed with reference to ISO 20502:2005 (ISO 20502 2005). The load at which complete removal of the coating occurs is usually taken as an indication of the adhesion strength. Further details on the mechanical testing methods of calcium orthophosphate coatings, films and layers might be found in literature (Ben-Nissan et al. 2011).

The adhesion strength of calcium orthophosphate coatings, films and layers depends on very many parameters. In the first instance, it strongly depends on the deposition technique. For example, HA coatings, obtained by PLD, showed greater adherence to a titanium alloy when compared with plasma-sprayed HA coatings (Vasanthan et al. 2008). Besides, it might depend on the coating thickness and its chemical composition, namely coatings of 50-μm thick gave higher values of the adhesion strength than those of 240-μm thick (Filiaggi et al. 1991), while scratch tests revealed that the sol-gel-fluorinated HA coating adhered to Ti-alloy substrate up to 35% better as the fluorine concentration increased in the coating (Zhang et al. 2006). Furthermore, the nature, structure and chemical composition of the substrate surface play an important role. For example, a highly roughened substrate surface exhibited higher bond strength as compared to a smooth substrate surface (Nimb et al. 1993). Besides, the adhesion strength of plasma-spayed coatings was found to decrease when either the plate power was reduced (from 28 to 22 kW) or the working distance was increased (from 90 to 130 mm) (Roy et al. 2011). Additionally, the bond strength of calcium orthophosphate coatings deposited on Ti plates pre-treated in an alkali solution followed by heat-treating (600°C for 1 h) in air had a higher value (approximately 35 MPa) if compared to those followed by heat-treating vacuum (approximately 21 MPa). This was attributed to the structural and compositional differences in the interfacial layer of sodium titanates (Wang et al. 2008c). For plasma-assisted deposition techniques of calcium orthophosphates, a good overview on the adhesion strength values of coatings, films and layers is presented in Table 3 of Surmenev (2012).

However, application of various inter-layers (synonym: buffer layers) seems to be the most important way to influence the adhesion strength of calcium orthophosphate coatings, films and layers to diverse substrates. A big number of the available deposition techniques (see ‘Preparation’ section above), which should be multiplied to a big selection of various substrates, result in a great number of potentially appropriate chemicals to be used as inter-layers between the substrates and calcium orthophosphates. For example, for plasma-assisted deposition methods of calcium orthophosphates, such chemicals as TiO_2_ (Rajesh et al. 2011), TiN (Nelea et al. 2000; Yang et al. 2009b, 2009c; Man et al. 2009), ZrO_2_ (Nelea et al. 2000) or Al_2_O_3_ (Nelea et al. 2000), were used as buffer layers. TiO_2_ (Nelea et al. 2007; Berezhnaya et al. 2010) and TiN (Nelea et al. 2003) were also used as under-layers for RF magnetron sputtering. Similarly, formation of intermediate layers of titanium hydroxides is required for biomimetic deposition of calcium orthophosphates on Ti (Wang et al. 2008a). To complicate things even further, one should mention, that mutual inter-diffusion of atoms, ions and molecules of calcium orthophosphates from coatings, films and layers from one side and those of a substrate from another side might occur. Especially, this is valid for high temperature deposition techniques; however, the mutual inter-diffusion might happen for any technique at the post-deposition annealing stage (see section ‘Brief knowledge on the important pre- and post-deposition procedures’). Various atomic mixed inter-layers are formed as the result. For example, the width (measured by Auger electron spectroscopy) of such inter-layer between a HA coating and magnesium substrate formed by IBAD technique was found to be approximately 3 μm (Yang et al. 2008). Such inter-layers can reduce the mismatch of thermal expansion coefficients between calcium orthophosphates and substrates, or increase the surface area of the material, wettability or heat conductivity; thus, increasing the bonding strength without affecting biocompatibility. Since the subject of inter-layers appears to be very broad, additional details are not specified further.

The adhesion forces depend on various factors, namely for dense coatings under tensile loading, failure usually occurs at the coating/substrate interface because the cohesive strength is higher than the bond strength. For porous coatings, the cohesive strength is low and the fracture occurs inside them (Han et al. 2001). The amorphous coatings have a more brittle nature and less adhesion compared to the crystalline ones (Cleries et al. 2000a). In general, the bond strength of apatite layer to Ti metal substrate is reported to range from 10 to 30 MPa (Kokubo et al. 1996; Kim et al. 1997). Similar values were obtained in another study, where calcium orthophosphate coatings were deposited on Ti substrates by a biomimetic method from two types of SBF. The results indicated that both the ionic concentrations of the SBFs and the surface roughness of the substrates had a significant influence on formation, morphology and bond strength of calcium orthophosphate precipitates. The highest bond strength of the precipitated coatings was about 15.5 MPa (Chen et al. 2009b).

#### Biodegradation

Biodegradation (synonyms: biotic degradation or biotic decomposition) is a chemical dissolution of materials by bacteria and/or other biological means. Since chemical composition of the body fluids might be considered as constancy, biodegradation of calcium orthophosphate coatings, films and layers is controlled by the properties of calcium orthophosphates themselves, which include their chemical composition, Ca/P ratio, crystal structure, crystallinity, porosity, lattice defects, particle sizes and purity (de Groot et al. 1998). For example, the dissolution kinetics of HA layers was studied using the dual constant composition method, and dissolution rates decreased when HA crystallinity increased (Tucker et al. 1996). Similar results were obtained in another study: after implantation, HA coatings with crystallinity of approximately 55% were found to degrade faster and possess better osteoinductivity than those with crystallinity of approximately 98% (Xue et al. 2005). Although biodegradation supposed to be *in vivo* process, various *in vitro* simulations are widely investigated. However, to be closer to the *in vivo* conditions, the biological assessments of calcium orthophosphate coatings, films and layers are performed in various simulating solutions, such as SBF (Kim et al. 2010; Chen et al. 2009b; Verestiuc et al. 2004; van der Wal et al. 2005, 2006; Heimann 2009; Łatka et al. 2010; d’Haese et al. 2010; Ntsoane et al. 2011), HBSS (Ueda et al. 2007; Man et al. 2009; Luo et al. 2000), aqueous saline solution (Surmenev et al. 2010; Ueda et al. 2009), Ringer’s solution (Gross and Berndt 1994; Gross et al. 1997), phosphate buffered saline (PBS) (Ueda et al. 2007; Boyd et al. 2006; Coelho et al. 2009a), Eagle’s minimum essential medium (Lim et al. 2005). Since the simulating solutions often contain dissolved ions of calcium and orthophosphates, both partial dissolution of calcium orthophosphate coatings, films and layers and their re-crystallization occurred (Verestiuc et al. 2004; Heimann 2009; d’Haese et al. 2010; Ntsoane et al. 2011; Lim et al. 2005). For example, as written in the abstract of d'Haese et al. (2010), ‘The soaking in SBF homogenizes the morphology of coatings. The sintered zone disappears, and the pores get filled by the reprecipitated calcium phosphates.’ One should stress that, due to the presence of other ions in the chemical composition of the aforementioned simulating solutions, in the vast majority of the cases not chemically pure but ion-substituted calcium orthophosphates are precipitated.

Usually, the biodegradation kinetics of calcium orthophosphate coatings, films and layers appears to be proportional to the solubility values of the individual ingredients, listed in Table 1. For example, both bone bonding and bone formation of HA, α-TCP and TTCP plasma-sprayed coatings were evaluated by mechanical push-out tests and histological observations after 3, 5, 15 and 28 months of implantation. Among them, α-TCP (which was the most soluble phase) showed the most significant degradation after approximately 3 months of implantation, while HA and TTCP showed significant signs of degradation only after approximately 5 months of implantation (Klein et al. 1994a). This resulted in lesser values of the mechanical push-out tests for α-TCP-coated implants if compared with those coated by HA and TTCP (Klein et al. 1991). Plasma-spray deposited coatings of HA were found to dissolve faster than the stoichiometric HA did because a high temperature melted HA powder and partly decomposed it into more soluble compounds, such as high-temperature ACP and OA (Pezeshki et al. 2010). Similarly, as-deposited magnetron spattered calcium orthophosphate coatings were almost amorphous (i.e., ACP), and therefore, they completely dissolved after exposure to PBS for only 24 h, while the dissolution rate of the same coatings after annealing (they became crystalline) was found to be more restrained (Boyd et al. 2006). Additionally, HA coatings were found to be less stable than those of FA (Klein et al. 1994b; Dhert et al. 1992; Dhert et al. 1993; Caulier et al. 1995) and of a similar stability with magnesium-whitlockite (*i.e.*, Mg-substituted β-TCP) coatings (Dhert et al. 1992, 1993).

On the other hand, there are cases (Gineste et al. 1999; de Bruijn et al. 1994; Cleries et al. 2000b), in which the biodegradation kinetics of calcium orthophosphate coatings, films and layers appeared to be correlated imperfectly with their solubility values (see Table 1). For example, three types of calcium orthophosphate (HA, ACP and β-TCP) coatings on titanium alloy substrates, deposited by the laser ablation technique, were immersed in SBF in order to determine their behavior in conditions similar to the human blood plasma. Neither HA nor ACP coatings were found to dissolve in SBF, while a β-TCP coating slightly dissolved. Precipitation of an apatitic phase was favored onto both HA and β-TCP coatings; however, no precipitation occurred onto ACP coating (Cleries et al. 2000b). Additionally, degradation rates of dental implants with 50- and 100-micron thick coatings of HA, FA and fluorhydroxylapatite (FHA) were studied (Gineste et al. 1999). The implants were inserted in dog jaws and retrieved for histological analysis after 3, 6, and 12 months. The thickness of the calcium orthophosphate coatings was evaluated using an image analysis device. HA and FA coatings (even at 100-micron thickness) were almost totally degraded within the implantation period. In contrast, the FHA coatings did not show significant degradation during the same period (Gineste et al. 1999).

#### Interaction with cells and tissue responses

The interactions of calcium orthophosphate coatings, films and layers with either cells *in vitro* or surrounding tissues *in vivo* have been studied a lot (Huang et al. 2009; Wang et al. 2004; Choi et al. 2003; Ueda et al. 2007; Massaro et al. 2001; Wie et al. 1998; Maistrelli et al. 1993; Hulshoff et al. 1996a; Caulier et al. 1997a, 1997b; Antonov et al. 1998; Cleries et al. 2000c; Lo et al. 2000; Jung et al. 2001; Manso et al. 2002c; Heimann et al. 2004; Siebers et al. 2004, 2005; Manders et al. 2006; Simank et al. 2006; Mello et al. 2007; Hashimoto et al. 2008; Coelho and Lemons 2009; Sima et al. 2010; Quaranta et al. 2010; Cairns et al. 2010). The *in vitro* trials using different cell lines revealed that in the vast majority of the cases, calcium orthophosphate coatings, films and layers enhanced cellular adhesion, proliferation and differentiation, while the results of the *in vivo* studies revealed that they promoted bone regeneration. For example, a combination of surface geometry and calcium orthophosphate coatings was found to benefit the implant-bone response during the healing phase (Hayakawa et al. 2002). Calcium orthophosphate coatings on titanium implants followed by bisphosphonate-immobilization appeared to be effective in the promotion of osteogenesis on surfaces of dental implants (Yoshinari et al. 2002). A greater percent of bone contact lengths were detected for calcium orthophosphate-coated Ti implants compared with control Ti implants 3 and 12 weeks after implant placement (Ong et al. 2002). Similar results were obtained in other studies (Dalton and Cook 1995; Nguyen et al. 2004; Yan et al. 2006).

Concerning the experiments with cells, human gingival fibroblasts attachment, spreading, extracellular matrix production and focal adhesion plaque formation were investigated on commercially pure Ti, HA-coated Ti and porous TCP/HA-coated Ti. TCP/HA and HA coatings exhibited that both the attached cell number and cell spreading area were higher than that on pure Ti and focal adhesion plaque formed earlier than that of uncoated substrate. The attached cell number and type I collagen formation on TCP/HA coatings were more than that on HA ones (Zhao et al. 2005). Osteoblasts were successfully grown on the surface of OCP (Bigi et al. 2005b) and HA (Cao et al. 2010a; Bigi et al. 2005b; Ball et al. 2001); both types of coatings, layers and films were found to favor osteoblast proliferation, activation of their metabolism and differentiation. Furthermore, the *in vitro* cell-culture studies using MG63 osteoblast-like cells were performed on calcium orthophosphate coatings deposited on titanium by plasma spray, sol-gel and sputtering techniques. The study demonstrated the ability of cells to proliferate on the materials tested. The sol-gel coating was found to promote higher cell growth, greater alkaline phosphatase activity and greater osteocalcin production compared to the sputtered and plasma-sprayed coatings (Massaro et al. 2001). In another study, calcium orthophosphate coatings were found to induce significantly higher cell differentiation levels than the uncoated control (Bucci-Sabattini et al. 2010).

A study by Cairns et al. (2010) should be described especially. Calcium orthophosphate thin films were deposited onto substrates with varying topography. Then, a layer of fibronectin was deposited from solution onto each surface, and the response of MG63 osteoblast-like cells was studied. The results revealed that, in all cases, the presence of the adsorbed fibronectin layer improved cell adhesion, proliferation and promoted early onset differentiation. Moreover, the nature and scale of the response appeared to be influenced by the surface topography of the substrates. Specifically, cells on the fibronectin-coated calcium orthophosphate thin films with regular topographical features in the nanometer range showed statistically significant differences in focal adhesion assembly, osteocalcin expression and alkaline phosphase activity compared to the calcium orthophosphate films without those topographical features (Cairns et al. 2010). Therefore, both an adsorbed bioorganics and a surface topography of the substrates appear to influence cell adhesion and differentiation.

*In vivo* results correlate well with the *in vitro* ones. Osseointegration rates of porous-surfaced Ti6Al4V implants with control (unmodified sintered coatings) were compared to porous-surfaced implants modified through the addition of either an inorganic or organic route sol-gel-formed calcium orthophosphate films. Implants were placed in distal femoral rabbit condyle sites and, following a 9-day healing period, implant fixation strength was evaluated using a pullout test. Both types of calcium orthophosphate films significantly enhanced the early rate of bone in-growth and fixation as evidenced by higher pullout force and interface stiffness compared with controls. However, there was no significant difference between calcium orthophosphate-coated implants prepared using the two different methods (Gan et al. 2004).

To conclude this section, one should note that the positive clinical benefits of calcium orthophosphate coatings, layers and films were not always detected. For example, a study was undertaken to evaluate the processes involved in biological responses of the Ti-6Al-7Nb alloy with and without HA coatings with both *in vitro* and *in vivo* tests. The results with HA coating appeared to be similar to those obtained on the uncoated samples (Lavos-Valereto et al. 2001). Similarly, neither positive nor negative influence of the presence of HA coatings on the surface of implants was detected during the 10-year (Lazarinis et al. 2011), 13-year (Camazzola et al. 2009), 15-year (Stilling et al. 2009) and undisclosed (Lee et al. 2000; Gandhi et al. 2009) follow-ups. Besides, there are cases in which short-term (4 weeks) advantages of the calcium orthophosphate-coated implants were found in animal studies, whereas no significant differences to the uncoated samples were found after 6 months (Gottlander et al. 1997a). Furthermore, inflammatory tissue reaction cases have been detected (Piattelli et al. 1995; Walschus et al. 2009). Interestingly, the short-term inflammatory response against a HA coating on Ti was lower in comparison to a DCPD coating on Ti. The observed differences between the Ti-DCPD implants and the Ti-HA implants were attributed to their dissolution characteristics: the HA coating on Ti showed increased stability and, hence reduced the inflammatory response (Walschus et al. 2009). Furthermore, HA coatings were found to be a risk factor for cup revision due to aseptic loosening (Lazarinis et al. 2010). Thus, precautions to prevent contamination (asepsis) and/or infection (perioperative antibiotics) appear to be more important for the calcium orthophosphate-coated implants if compared with the uncoated ones (Oosterbos et al. 2002).

### Biomedical applications

**Figure 11:**
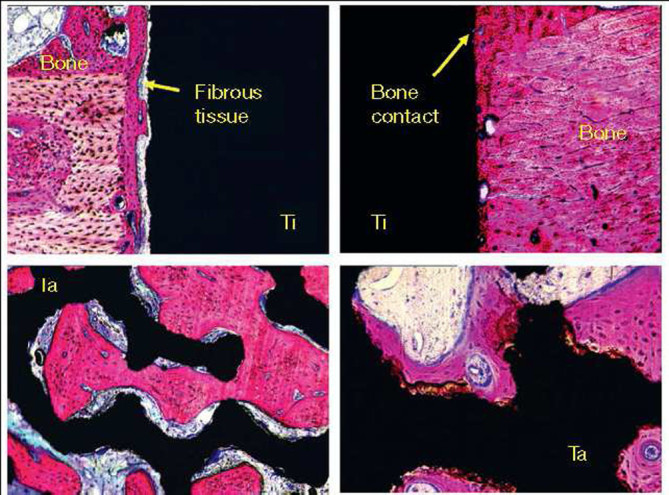
**Comparison of bone-integrative properties.** Non-coated (left) and biomimetically coated by calcium orthophosphates metal implants (right) after implantation in the femur of goats for 6 weeks. Reprinted from Layrolle (2011) with permission.

Already in 1987, de Groot et al. (1987), published a paper on the development of plasma-sprayed HA coatings on metallic implants. The same year the same researchers published the results of the first clinical study (Geesink et al. 1987). Shortly afterwards, Furlong and Osborn, two leading surgeons in the orthopedics field, began implanting plasma-sprayed HA stems in patients (Furlong and Osborn 1991) followed by other clinicians (Bauer et al. 1991; Buma and Gardeniers 1995). Since then, plentiful reports have been published about the biomedical advantages of such coated implants. To summarize the available information on the biomedical and biomechanical properties of implants coated by calcium orthophosphates, one can claim the following: If compared to uncoated implants, the presence of calcium orthophosphate deposits were found to induce bone contacts to the implants (Dhert et al. 1992, 1993; Thomas et al. 1989; Jansen et al. 1991; Gottlander et al. 1997b; Hulshoff and Jansen 1997; Hayakawa et al. 2000; Mohammadi et al. 2004; Park et al. 2005; Siebers et al. 2007; Kuroda et al. 2007; Chae et al. 2008; Schwarz et al. 2009; Junker et al. 2010; Suzuki et al. 2010); improve implant fixation (Yang et al. 1997; Søballe et al. 1993; Daugaard et al. 2010); show higher torque values (Park et al. 2005; Junker et al. 2010; Granato et al. 2009) and push-out strength (Ozeki et al. 2001); facilitate bridging of small gaps between implants and surrounding bones (Søballe et al. 1991; Stephenson et al. 1991), reduce metal ion release from the metallic substrates (Surmenev et al. 2010; Ducheyne and Healy 1988; Sousa and Barbosa 1996; Ozeki et al. 2003); slow down metal degradation and/or its corrosion (Metikoš-Huković et al. 2003; Yang et al. 2008; Cheng and Roscoe 2005); accelerate bone growth (Cook et al. 1992; Wang et al. 2009), remodeling (Pilliar et al. 1991; Yoon et al. 2009) and osteointegration rate (Bigi et al. 2008; Lee et al. 2011); induce osteoconductivity (Cao et al. 2010b), improve the early bone (Yang et al. 1996; Mohammadi et al. 2003) and healing (Vercaigne et al. 2000b) responses; and result in lack of formation of fibrous tissues (Figure 11) (Layrolle 2011; Dostálová et al. 2001), as well as increase the clinical performance of orthopedic hip systems (see below). In addition, calcium orthophosphate coatings, films and layers might be used for incorporation of drugs and important biologically active compounds, such as peptides, hormones and growth factors (Siebers et al. 2006). In the case of porous implants, calcium orthophosphate coatings enhance bone ingrowth into the pores (Suchanek and Yoshimura 1998). Furthermore, studies concluded that there was significantly less pin loosening in calcium orthophosphate-coated groups (Saithna 2010). Thus, the majority of the clinical studies are optimistic about the *in vivo* performance of calcium orthophosphate-coated prostheses. However, to be objective, one must mention on the studies in which no positive biomedical and/or biomechanical effects of calcium orthophosphate coatings, films and layers have been detected (Tieanboon et al. 2009; Coelho et al. 2009b). Besides, the presence or absence of the positive biomedical and/or biomechanical effects of calcium orthophosphate coatings, films and layers might depend on the deposition technique used (Hulshoff et al. 1996b, 1997), as well as on the coating vendor (Dalton and Cook 1995). These uncertainties might be due to several reasons, such as variability in chemical and phase composition, porosity, admixtures, *etc.*

**Figure 12:**
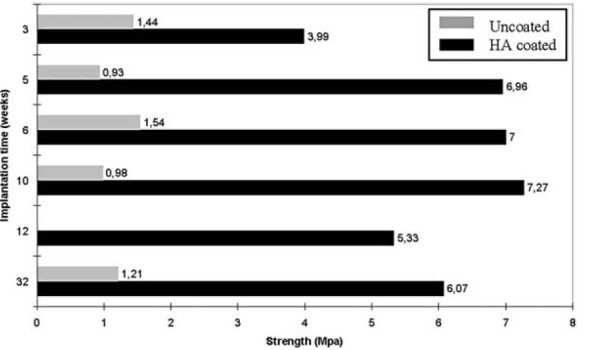
**Time-dependent plasma-sprayed HA coating.** This figure shows how a plasma-sprayed HA coating on a porous titanium (dark bars) dependent on the implantation time will improve the interfacial bond strength compared to uncoated porous titanium (light bars). Reprinted from Hench (1991) with permission.

In biomedical applications, bone grafts are usually much thicker than coatings, films or layers applied to them. Nevertheless, the coated implants combine the surface biocompatibility and bioactivity of calcium orthophosphates with the core strength of strong substrates (Figure 12). The clinical results for calcium orthophosphate-coated implants reveal that they have much longer life times after implantation than uncoated devices, and therefore, they are particularly beneficial for younger patients (Capello et al. 1997). Their biomedical properties are approaching those of bioactive glass-coated implants (Wheeler et al. 2001; Mistry et al. 2011).

Since, among calcium orthophosphates, HA is the most popular material to be deposited as coatings, films and layers, the vast majority of the clinical investigations was performed with HA. For example, HA coating as a system of fixation of hip implants *in vivo* was found to work well in the short to medium terms (2 years (Geesink 1990), 6 years (Geesink and Hoefnagels 1995), 8 years (Wheeler 1996; Chang et al. 2006), 9 to 12 years (MaNally et al. 2000), 10 years (Oosterbos et al. 2004; Trisi et al. 2005), 10 to 15.5 years (Matsumine et al. 2004), 10 to 17 years (Muirhead-Allwood et al. 2010), 13 to 15 years (Shetty et al. 2005), 15 to 21 years (Rajaratnam et al. 2008), 16 years (Buchanan 2005), 17 years (Buchanan 2006) and 19 years (Buchanan and Goodfellow 2008)). In 2004, a special book summarizing the studies with HA-coated implants and the ‘state of the art’ of HA coatings in orthopedics at the close of 2002 was published (Epinette and Manley 2004). Similar data for HA-coated dental implants are also available (Tinsley et al. 2001; Binahmed et al. 2007; Iezzi et al. 2007). Nevertheless, even longer-term clinical results are awaited with a great interest. The biomedical aspects of osteoconductive coatings for total joint arthroplasty have been reviewed elsewhere (Geesink 2002). Additional details on calcium orthophosphate coatings, films and layers might be found in excellent reviews (Narayanan et al. 2010; Paital and Dahotre 2009a; León and Jansen 2009).

Nevertheless, one must stress that although many experiments concerning the *in vivo* studies of calcium orthophosphate coatings, films and layers have indicated a stronger and faster fixation, as well as more bone ingrowth at the interface, the clinical performance of such coatings, films and layers is still far from the perfection. Some of the major concerns associated with the usage of calcium orthophosphate coatings, films and layers in actual body environment, with regard to their long-term stability can be listed as follows (Hulshoff et al. 1996a; Caulier et al. 1997a, 1997b):

- The degradation and resorption of calcium orthophosphate coatings, films and layers in a biological environment could lead to disintegration of the coating, resulting in the loss of both coating-substrate bond strength and the implant fixation.
- Coating delamination and disintegration with the formation of particulate debris are also major concern
- Calcium orthophosphate coatings, films and layers may also lead to increased polyethylene wear from the acetabular cup and, thereby, alleviate the problem of osteolysis.

In spite of the long history and the aforementioned achievements, still not all concerns on the surgical applications of calcium orthophosphate coatings, films and layers have been eliminated. Still, a limited amount of the *in vivo* studies is available in the literature. The limitations to such experiments may be attributed to any of the following reasons:

- Difficulty in selection of a suitable animal model to simulate the actual mechanical loading and unloading conditions the implant might undergo in a human body environment.
- The need to sacrifice a large number of animals, since most of these experiments demand a statistical analysis to validate the results.
- A high cost and a long period of clinical testing these experiments demand.
- Lack of coordination among material scientists and biologists and thereby an insufficient understanding of this interdisciplinary subject.
- Serious ethical concerns on the use of animals for experimental studies as they are subjected to painful procedures or toxic exposures during the course of test.

To conclude this section, one must note the following: Even though the importance and the need for development of calcium orthophosphate coatings, films and layers have been recognized, it is still mostly being explored on research level, and after extensive search of open literature, these coatings appear to have made limited headway into commercialization. In spite of mention of the commercial products such as hip and dental implants produced by Zimmer Orthopedics (Freiburg, Germany), Smith and Nephew (Memphis, TN, USA), and Biomet, the science and technology related to their manufacturing is not disclosed by any one of them due to the proprietary reasons. Hence, at this point it is difficult to bring a detailed discussion on commercialization of calcium orthophosphate coatings, films and layers (Paital and Dahotre 2009a).

### Future directions

A potential drawback of the majority of the deposition techniques of calcium orthophosphate coatings, films and layers is their relatively high cost for a large scale production. Therefore, to decrease processing time and make their manufacturing commercially viable, it is desirable to process the thinnest coating that would significantly increase the biological response (Coelho et al. 2009c). Much attention should be paid to functionally graded structures with an amorphous top layer and a crystalline layer underneath (Wang and Zreiqat 2010). This allows adjusting the coating resorption rates to the values at which new bone grows at early stages when it is of the most importance for the bone mineralization process. Furthermore, therapeutic capabilities of calcium orthophosphate coatings, films and layers as templates for the *in situ* delivery of drugs and osteoinductive agents (peptides, hormones and growth factors) at the required times should be elucidated much better.

## Conclusions

Solid implants prepared from various materials often possess a poor biocompatibility with a simultaneous lack of the osteogenic properties in order to promote bone healing. In addition, direct bone-to-implant contacts are desired for a biomechanical anchoring of implants rather than fibrous tissue encapsulation. All these problems might be solved by applying calcium orthophosphate coatings, films and layers. The aim is to provide the implants with surface biological properties for adsorption of proteins, adhesion of cells and bone apposition. Therefore, the available knowledge on calcium orthophosphate and, most notably, HA coatings, films and layers on various substrates has been summarized in this review. Since all available deposition techniques have both advantages and shortcomings of their own (Table 3), still there are no standard guidelines for depositing calcium orthophosphates on the implant surfaces. In general, dissolution of calcium orthophosphate coatings, films and layers improves implant osseointegration and is the basic requirement for bioactivity. However, this dissolution diminishes the stability and increases the potential for loosening of the implants. Calcium orthophosphate coatings, films and layers of lower solubility and higher stability are desirable for the long-term performance of implants because they promote faster initial bone fixation, bridge larger gaps in the misfit and degrade at a controlled rate.

Although animal and *in vitro* studies have already reported on the benefits of using calcium orthophosphate-coated implants, as well as the risks of dissolution, the short-term studies did not demonstrate that the dissolution of calcium orthophosphate coatings, films and layers led to a loss of implants. In addition, many *in vivo* and clinical studies did not consider the chemical and structural characterizations of the coatings. Under these conditions, any comparisons among various reports and studies are difficult.

New promising techniques for coating medical devices are continuously investigated. Future investigations on various coating processes will have to include clinical trials to get better understanding of bone responses to coated-implant surfaces, as well as studies on coupling of calcium orthophosphate coatings, layers and films with drugs, growth factors and cells. Although it has been generally accepted that calcium orthophosphate coatings, layers and films improve bone strength and initial osteointegration rate, the optimal coating properties required to achieve maximal bone response are yet to be reported. As such, the use of well-characterized calcium orthophosphate coatings, layers and films cell culture studies, animal studies and clinical studies should be well documented to avoid controversial results.

In addition, the clinicians need to take into consideration the enhanced bacterial susceptibility of calcium orthophosphate-coated implants if compared to the metallic ones. Besides, the clinicians need to consider possible failures of calcium orthophosphate coatings, films and layers as a result of coating-substrate interfacial fracture. It is also important that the clinical investigators be well versed with the material characterizations of the coated implants.

## Authors’ original submitted files for images

Below are the links to the authors’ original submitted files for images. Authors’ original file for figure 1 Authors’ original file for figure 2 Authors’ original file for figure 3 Authors’ original file for figure 4 Authors’ original file for figure 5 Authors’ original file for figure 6 Authors’ original file for figure 7 Authors’ original file for figure 8 Authors’ original file for figure 9 Authors’ original file for figure 10 Authors’ original file for figure 11 Authors’ original file for figure 12 Authors’ original file for figure 13
