# Supplementary figures and images for: Calcium orthophosphate coatings, films and layers
Source: Prog Biomater. 2012 Sep 26;1:1. doi: 10.1186/2194-0517-1-1 (PMC5120666; doi:10.1186/2194-0517-1-1)

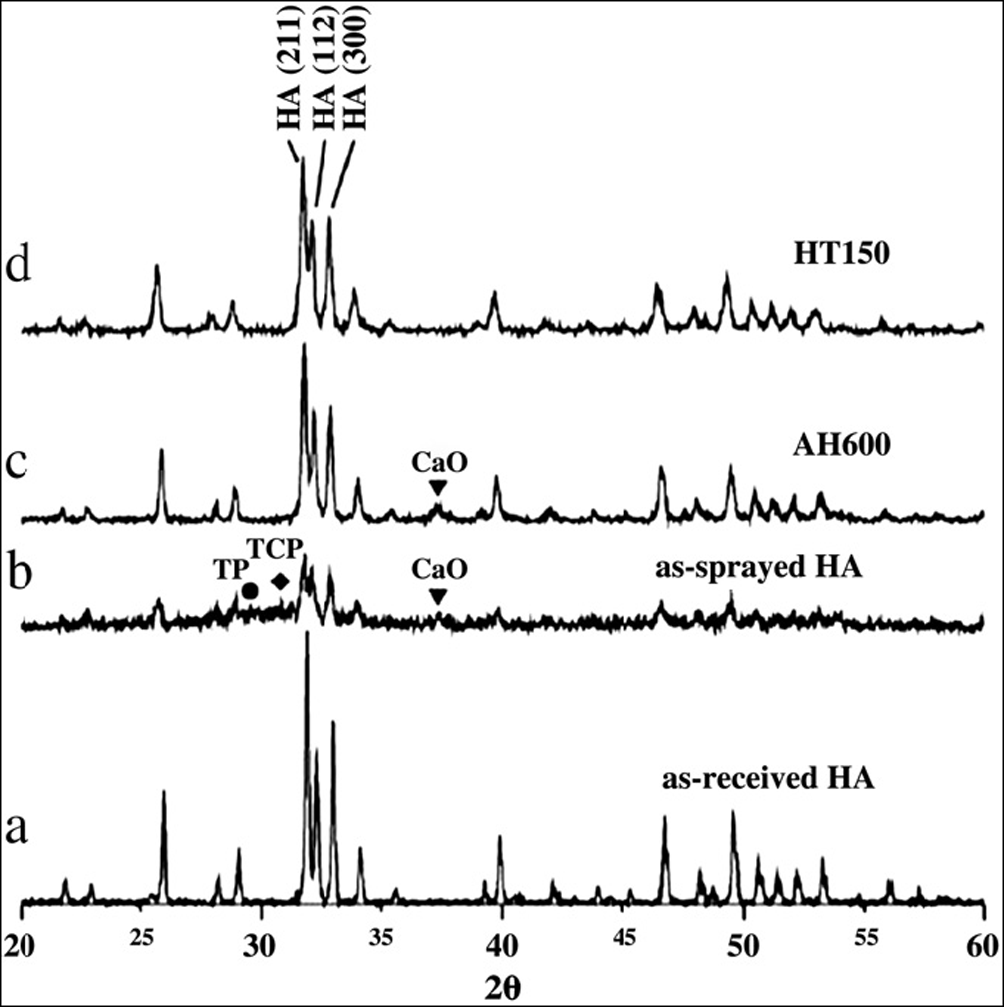

Supplement: Supplementary file 1 — Authors’ original file for figure 1 [file 40204_2012_1_MOESM1_ESM.tiff]

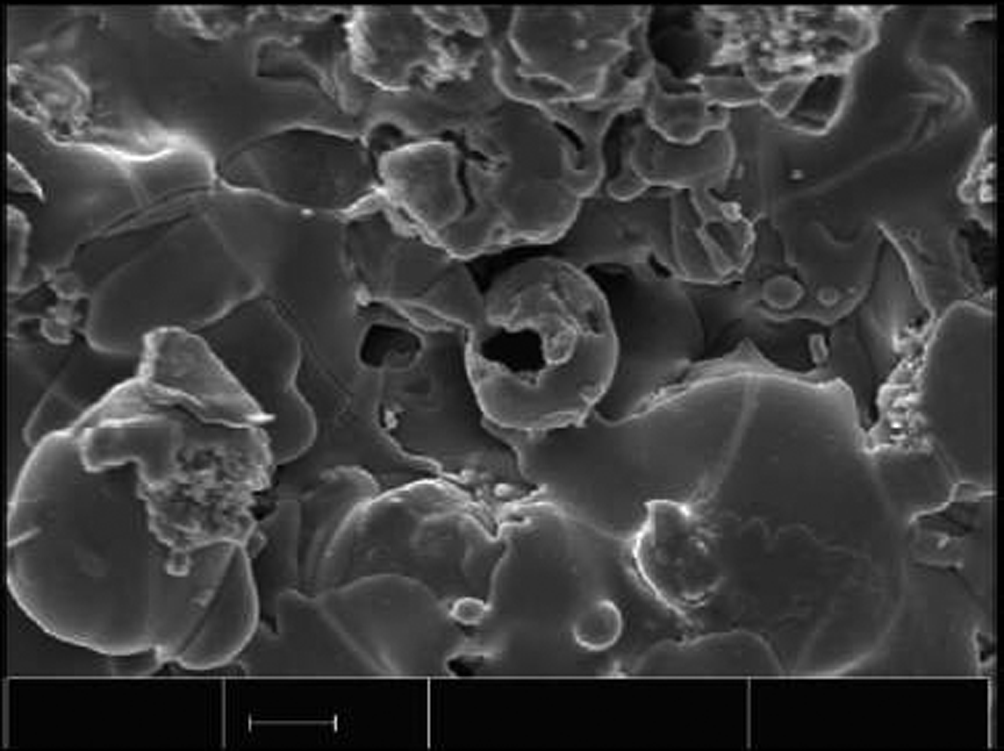

Supplement: Supplementary file 2 — Authors’ original file for figure 2 [file 40204_2012_1_MOESM2_ESM.tiff]

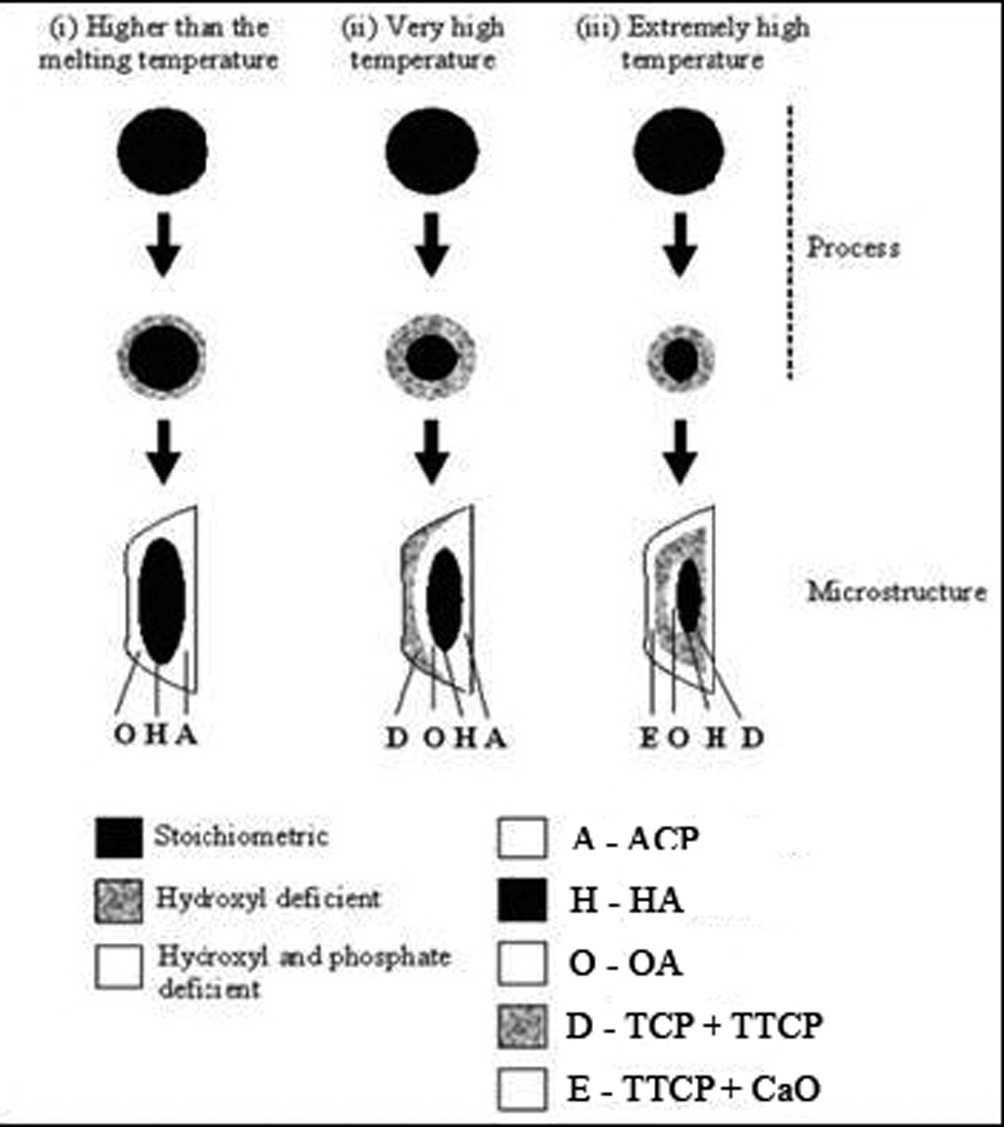

Supplement: Supplementary file 3 — Authors’ original file for figure 3 [file 40204_2012_1_MOESM3_ESM.tiff]

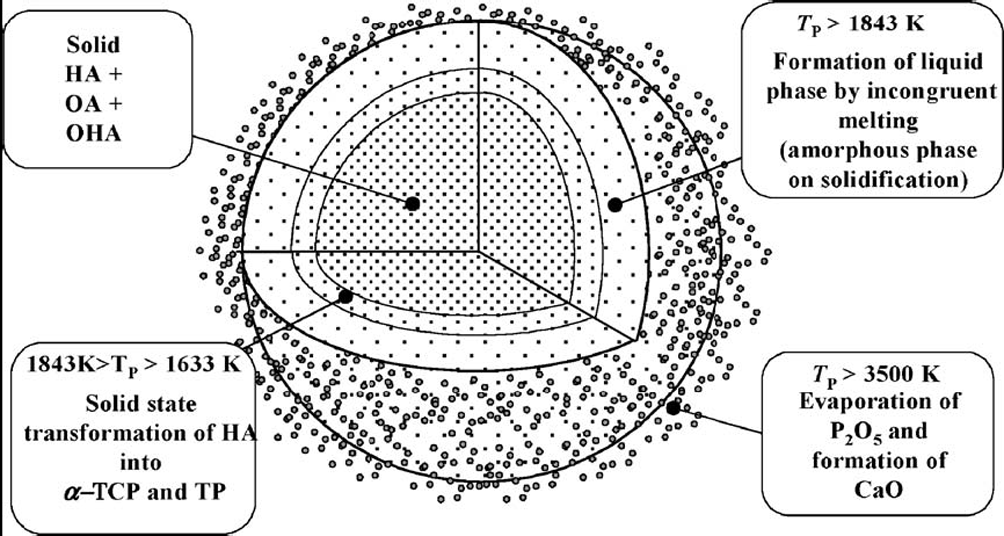

Supplement: Supplementary file 4 — Authors’ original file for figure 4 [file 40204_2012_1_MOESM4_ESM.tiff]

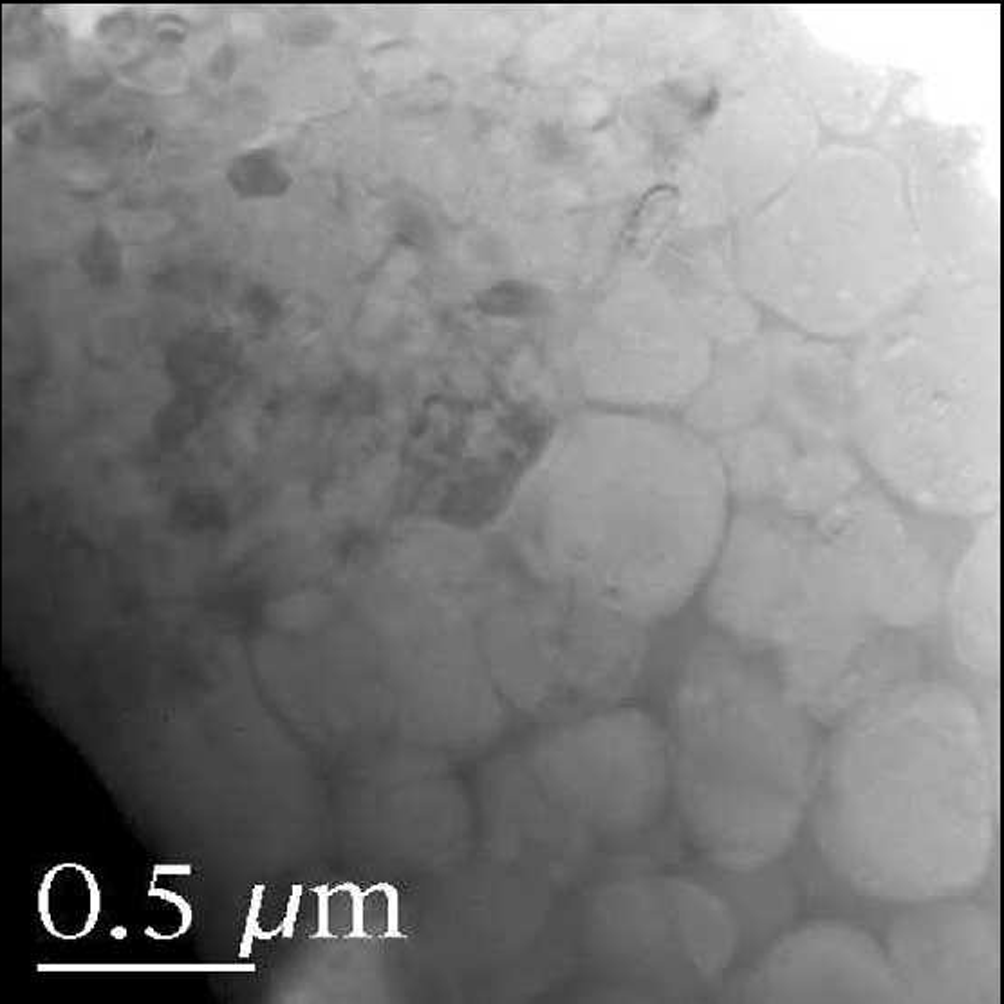

Supplement: Supplementary file 5 — Authors’ original file for figure 5 [file 40204_2012_1_MOESM5_ESM.tiff]

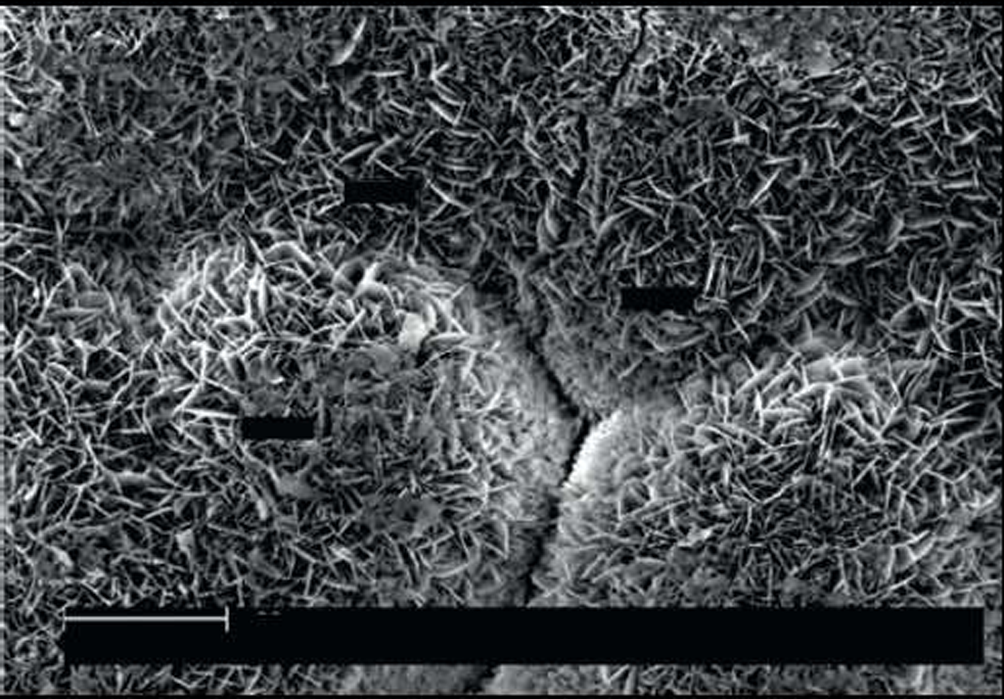

Supplement: Supplementary file 6 — Authors’ original file for figure 6 [file 40204_2012_1_MOESM6_ESM.tiff]

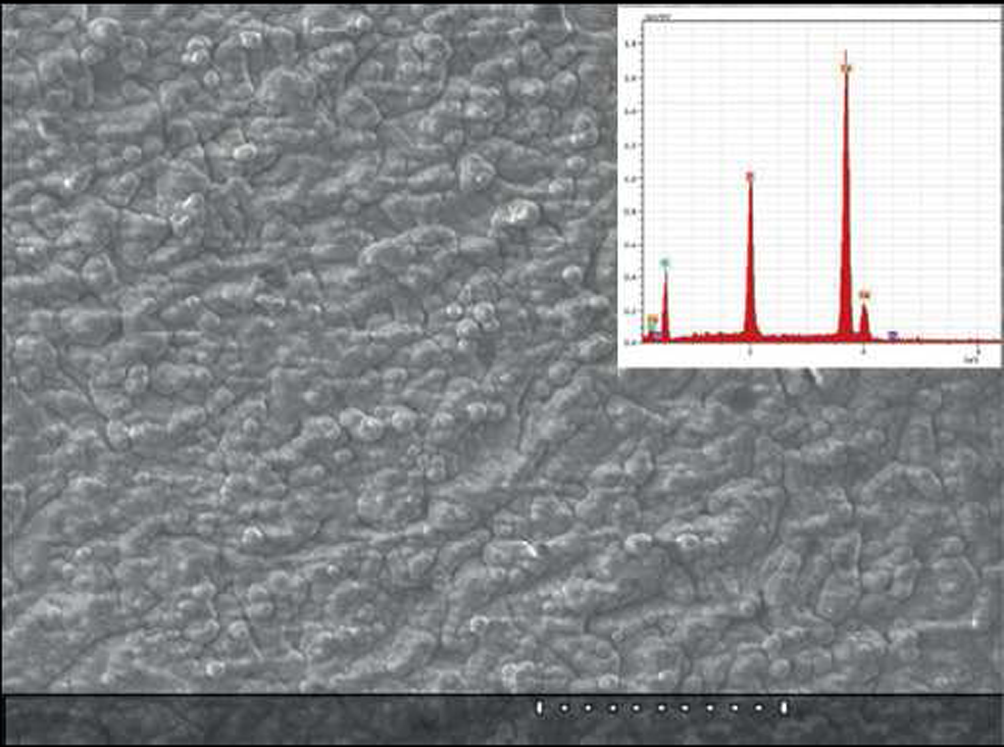

Supplement: Supplementary file 7 — Authors’ original file for figure 7 [file 40204_2012_1_MOESM7_ESM.tiff]

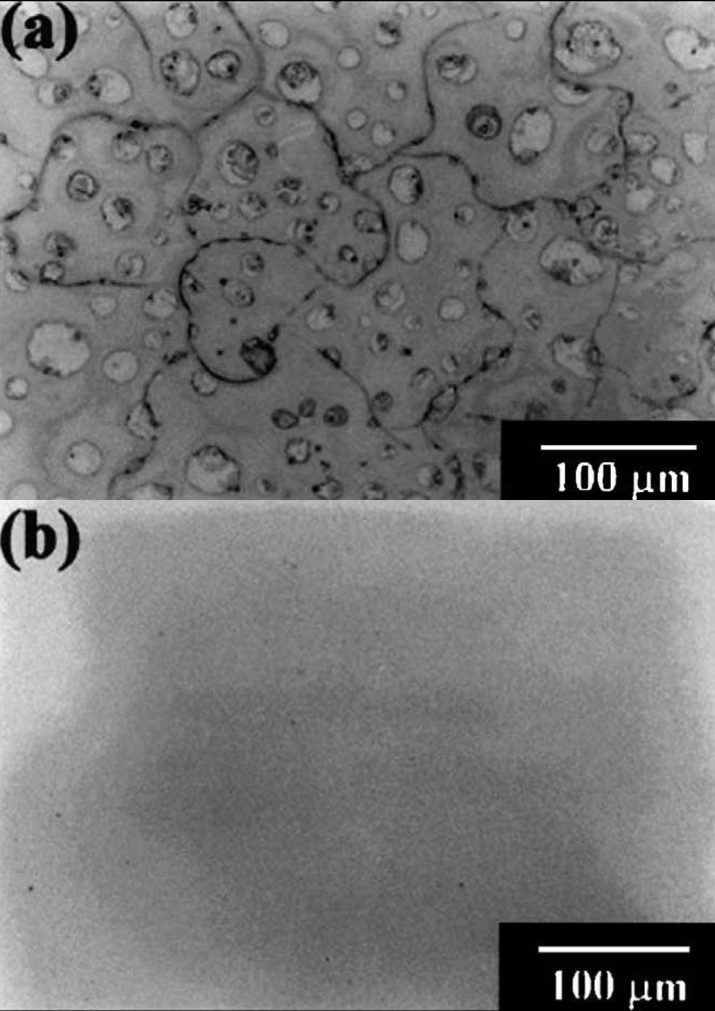

Supplement: Supplementary file 8 — Authors’ original file for figure 8 [file 40204_2012_1_MOESM8_ESM.tiff]

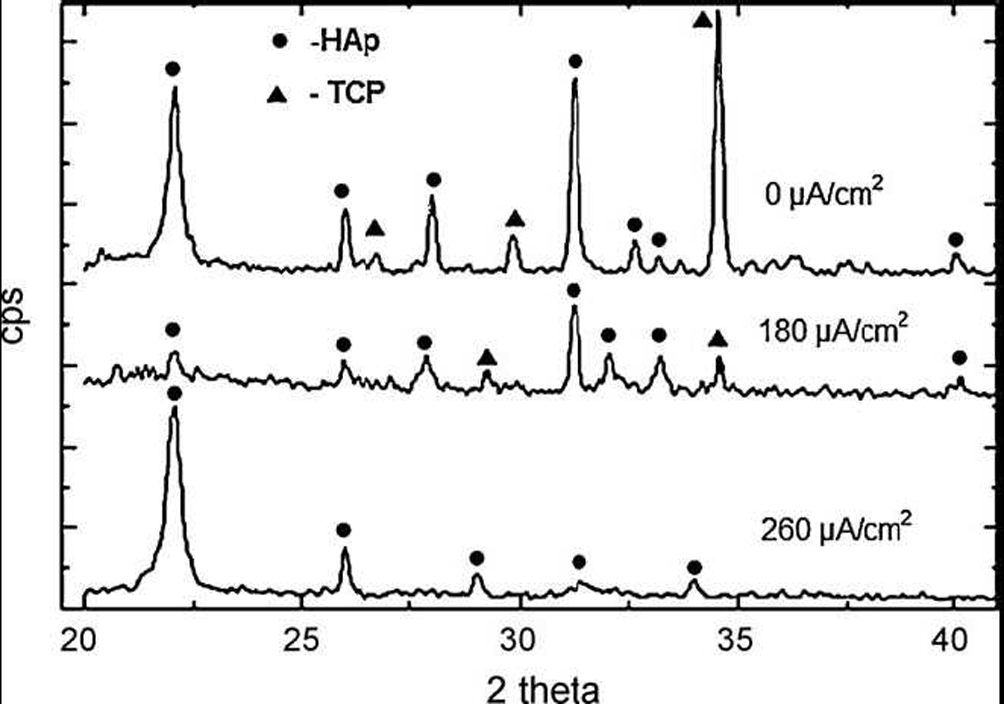

Supplement: Supplementary file 9 — Authors’ original file for figure 9 [file 40204_2012_1_MOESM9_ESM.tiff]

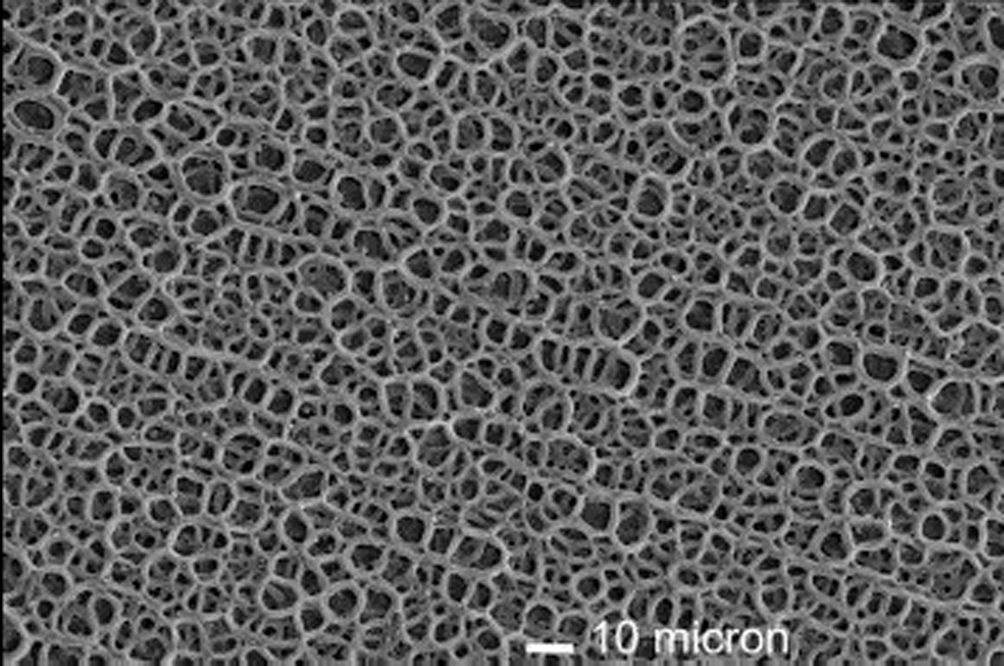

Supplement: Supplementary file 10 — Authors’ original file for figure 10 [file 40204_2012_1_MOESM10_ESM.tiff]

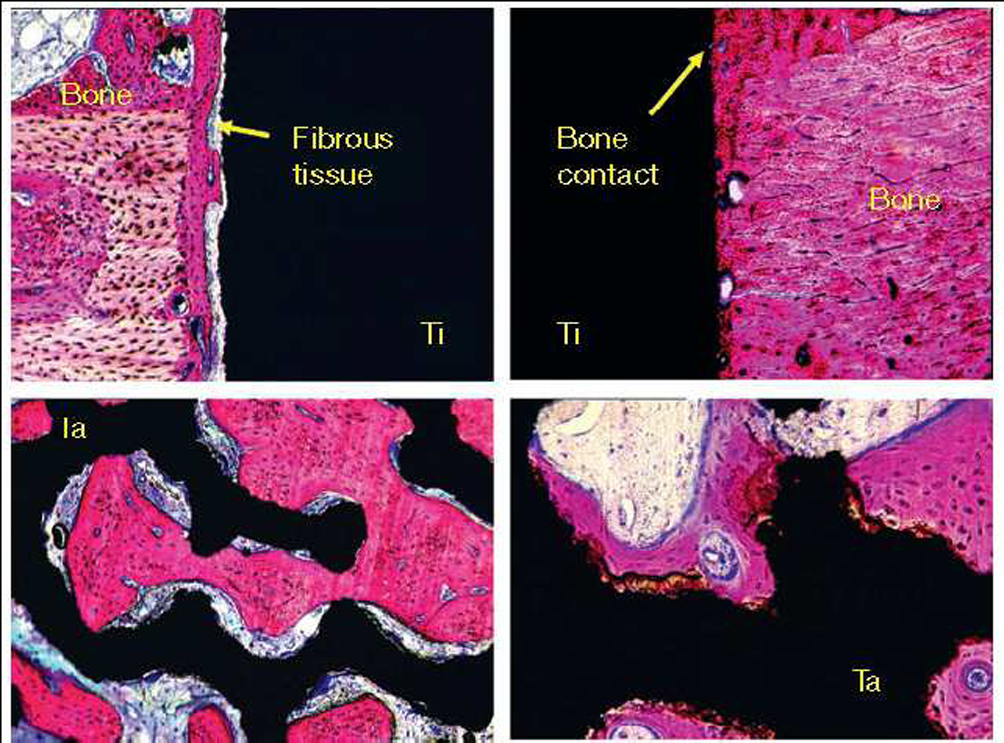

Supplement: Supplementary file 11 — Authors’ original file for figure 11 [file 40204_2012_1_MOESM11_ESM.tiff]

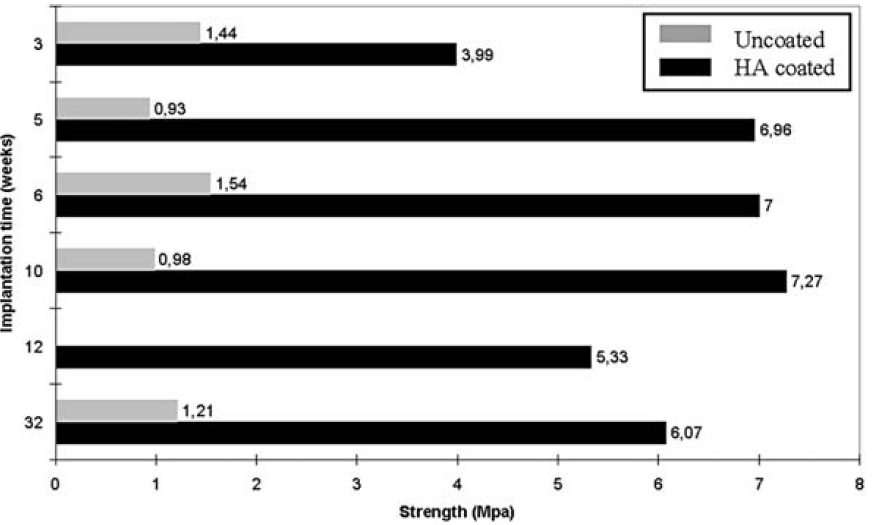

Supplement: Supplementary file 12 — Authors’ original file for figure 12 [file 40204_2012_1_MOESM12_ESM.bmp]

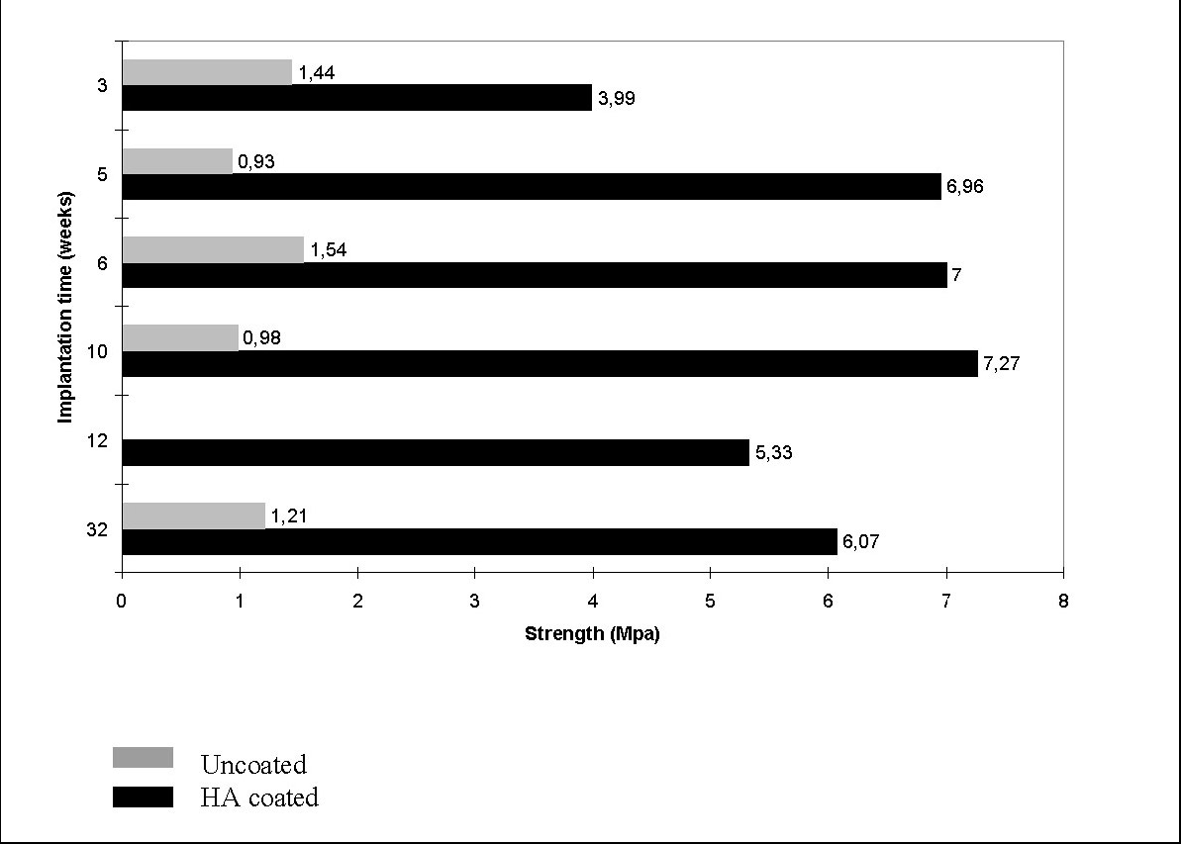

Supplement: Supplementary file 13 — Authors’ original file for figure 13 [file 40204_2012_1_MOESM13_ESM.tiff]
